# AFF3 maintains metabolic quiescence in naive CD8 T cells and prevents premature immune aging

**DOI:** 10.1172/jci.insight.207387

**Published:** 2026-06-23

**Authors:** Molly E. Lumnitzer, Stefanie F. Valbon, Stephanie A. Condotta, Allison E. Norlander, Sheng Liu, Jun Wan, Martin J. Richer

**Affiliations:** 1 Department of Microbiology and Immunology, IU Cooperative Center of Excellence in Hematology, and; 2 IU Melvin and Bren Simon Comprehensive Cancer Center, Indiana University School of Medicine (IUSM), Indianapolis, Indiana, USA.; 3 Département de Microbiologie, Immunologie et Infectiologie, Université de Montréal, Centre de Recherche de l’Hôpital Maisonneuve-Rosemont, Montréal, Quebec, Canada.; 4 Rosalind and Morris Goodman Cancer Institute, McGill University, Montreal, Quebec, Canada.; 5 Department of Anatomy, Cell Biology & Physiology,; 6 Department of Medicine, and; 7 Department of Medical & Molecular Genetics, IUSM, Indianapolis, Indiana, USA.

**Keywords:** Aging, Immunology, Metabolism, Glucose metabolism, Metabolomics, T cells

## Abstract

It is necessary for naive CD8 T cells to be actively maintained in a quiescent metabolic state in order to respond robustly to infection while avoiding inappropriate activation during homeostasis. With age, this quiescent state is lost and the CD8 T cell response to infection decreases. The factors regulating metabolic quiescence of CD8 T cells and how this regulation is lost during aging are not completely understood. Herein, we identify the transcription factor AFF3 as a regulator of metabolic quiescence in naive CD8 T cells. While naive AFF3-deficient CD8 T cells are more metabolically active prior to infection, they have reduced accumulation in response to viral infection, and this is correlated with a poor capacity to engage glycolysis. During aging in both murine and human CD8 T cells, AFF3 expression is decreased. In mice, this is associated with a loss of metabolic quiescence and reduced capacity to accumulate following infection. Our data highlight the role of metabolic regulation in CD8 T cell quiescence and identify a transcription factor that may be a target to reinvigorate CD8 T cell responses during aging.

## Abstract 2

AFF3 is important to the function of cytotoxic T cells by regulating their metabolism. This protein is also important to prevent premature immune aging.

## Introduction

Activated CD8 T cells recognize and eliminate virally infected or cancerous cells in a cognate antigen specific manner (1, 2). These cells patrol secondary lymphoid organs until they are activated (3). CD8 T cells are actively maintained in a quiescent state to prevent immunopathology that could result from premature activation (4–8). Recent studies have shown that maintaining quiescence of naive CD8 T cells is also essential for robust immune responses upon activation (4, 5, 7–9). Maintenance of quiescence is complex and involves multiple distinct and complementary mechanisms, including the regulation of T cell genomic architecture by LEF-1 and TCF-1 interactions (10, 11) and BACH 2 and SATB1 loop formations (12). Additionally, the maintenance of a quiescent metabolism is critical to maintain T cell responsiveness (13–15). This includes the regulation of mTORC1, a central player in cellular metabolism that integrates multiple signaling pathways and environmental cues (13, 16–18). For example, the Raptor-mTORC1 signaling pathway is critical for T cells to exit quiescence, and Raptor deficiency leads to activation and proliferation defects in T cells (9). In addition, premature activation of mTORC1 and exit from T cell quiescence prior to activation is detrimental to the generation of protective immune responses (9). This is highlighted by the importance of tumor suppressing gene Tuberous Sclerosis Complex (TSC1) in regulating mTORC1 activity in naive T cells to maintain their quiescence and prevent hyperactivation (7). Lack of TSC1 leads to a loss of quiescence in naive T cells, which causes T cells to respond poorly to infection (7). Finally, the cytokine milieu encountered by quiescent CD8 T cells can affect their ability to respond to viral infection, with inflammatory cytokines playing an important role in naive T cells exiting quiescence, a pathway that depends on the balance between Stat1 and Stat4 signaling (19, 20). Together, these data show that the active regulation of T cell quiescence is required to prevent inappropriate activation and to maintain optimal responses to infection.

During aging, CD8 T cells are exposed to increased systemic inflammation, termed inflammaging, which can cause T cells to lose quiescence and become less responsive to infection (5, 21–26). Therefore, understanding the regulation of sustained CD8 T cell quiescence is important as a possible mechanism to prevent aging-associated T cell dysfunction. In aged individuals, naive CD8 T cells increase lipid metabolism, leading to increased apoptosis and reduced proliferation during immune responses (27). This further supports the idea that quiescence, including metabolic quiescence, of naive T cells affects their responses to pathogens. In this study, we found that the transcription factor AFF3, an AF4/FMR2 family member with no previously described role in CD8 T cells, helps maintain a quiescent metabolism in naive T cells and to respond optimally to infection. Importantly, our data suggest that AFF3 regulates naive CD8 T cell quiescence independently of acquiring an activated or memory-like phenotype. Finally, we observed that *Aff3* expression is diminished in CD8 T cell during aging, and this reduction is associated with T cells developing a premature aged-like metabolic phenotype.

## Results

### The expression of Aff3 is downregulated in CD8 T cells activated in the presence of inflammatory cytokines.

**Figure 1:**
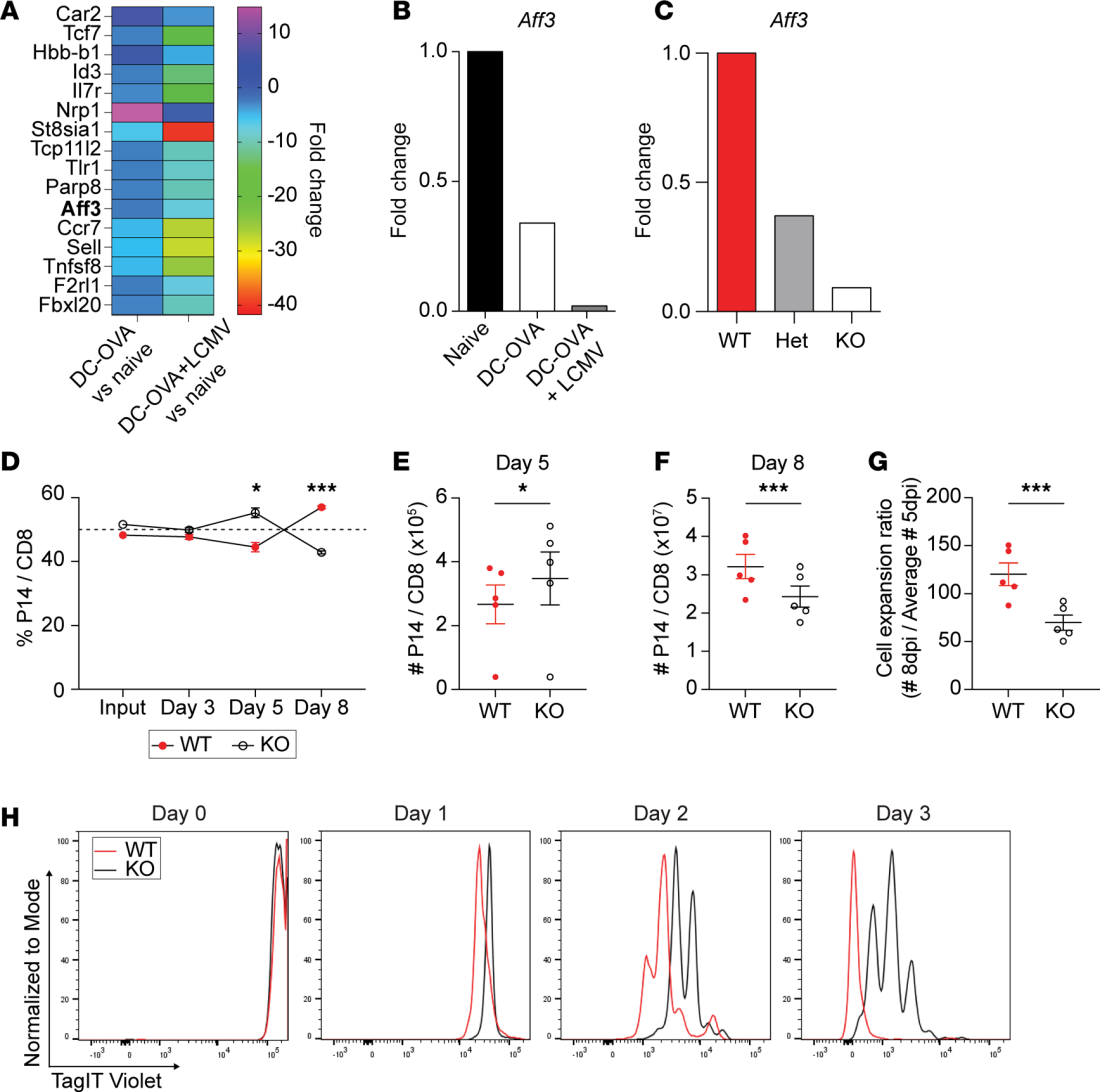
Aff3 is decreased with inflammation and necessary for a robust T cell response to viral infection. (**A**) Heatmap of the top 15 differentially expressed genes in OT-I CD8 T cells from the spleen of DC-OVA or DC-OVA + LCMV immunized mice on day 5 compared with naive OT-I CD8 T cells identified by microarray. (**B**) Relative mRNA expression of *Aff3* from OT-I CD8 T cells isolated from the spleen of DC-OVA or DC-OVA + LCMV immunized mice by RT-qPCR analysis compared with normalized expression in naive OT-I CD8 T cells. (**C**) Relative mRNA expression of *Aff3* from CD8 T cells isolated from the spleens WT, AFF3 heterozygous (Het), and AFF3 KO mice by RT-qPCR analysis. (**D**) Frequency of splenic WT or AFF3 KO P14 CD8 T cells at input, or from the spleen 3 dpi, 5 dpi, and 8 dpi with LCMV Arm. (**E** and **F**) Number of WT or AFF3 KO P14 CD8 T cells in the spleen at 5 dpi and 8 dpi with LCMV Arm. (**G**) Expansion ratio of WT and AFF3 KO P14 CD8 T cells from 5 to 8 dpi with LCMV Arm. (**H**) Representative histogram plots of the dilution of TagIT Violet proliferation dye by splenic WT and AFF3 KO CD8 T cells on days 0–3 of ex vivo culture. (**A**) Pooled CD8 T cells from 1–3 mice with 3 replicates per condition and representative of at least 2 separate experiments. (**B** and **C**) Pooled CD8 T cells from 1–3 mice in each condition. (**D**–**H**) Representative of 2 experiments with *n* = 3–5 mice per experiment. (**D**–**G**) Groups compared using paired Student’s *t* test, *P < 0.05, ***P < 0.001. Data represent mean ± SEM.

Dynamic changes occur in CD8 T cells when activated in the presence of inflammatory cytokines, including changes in the regulation of their quiescence (19, 20). Changes to quiescent naive cells are critical to regulate the expansion, function, differentiation, trafficking, and metabolism of activated cells (28). To determine genes regulated in activated T cells by inflammatory cytokines, we used a previously described in vivo dendritic cell (DC) immunization approach (29). Mice were adoptively transferred with OT-I CD8 T cells (T cell receptor [TCR] transgenic CD8 T cells specific to ovalbumin [OVA]_257-264_) and immunized with LPS-matured DCs loaded with OVA_257-264_ peptide (DC-OVA), or LPS-matured DCs loaded with OVA_257-264_ peptide and lymphocytic choriomeningitis virus (LCMV) infection (DC-OVA + LCMV). In this approach, LCMV infection provides a source of inflammatory cytokines without providing antigenic stimulation for the OT-I CD8 T cells, which do not respond to LCMV antigens, allowing us to separate the effect of inflammatory cytokines on T cells from the effects of TCR and costimulation. Transcriptomic changes were analyzed using microarray. One of the downregulated genes in the presence of systemic inflammation was *Aff3* (Figure 1A). This transcription factor is highly expressed in lymphoid tissues and plays an important role in class switch recombination in B cells (30). Additionally, genome-wide association studies (GWAS) have shown that high expression of *AFF3* is a potential risk factor in autoimmune conditions such as Rheumatoid arthritis (31). To date, AFF3 has no defined role in CD8 T cells, despite being expressed by naive CD8 T cells. Microarray results were confirmed via RT-qPCR, which demonstrated that while immunization with DC-OVA (signal 1 and 2 only) led a to reduction in *Aff3* expression compared with naive OT-I CD8 T cells, expression was further reduced by inflammatory cytokines induced by infection with LCMV (Figure 1B). Together, these data establish that *Aff3* expression is dynamically regulated, downregulated by T cell activation, and further reduced by inflammatory cytokines. This suggests that AFF3 may be a negative regulator of T cell activation and differentiation.

To study the role of AFF3 in CD8 T cell responses to viral infection, we generated conditional KO mice by crossing *Aff3*^fl/fl^ mice with CD4-Cre mice to create *Aff3*^fl/fl^ CD4-Cre^+^ (AFF3-KO) mice in which AFF3 is deleted in all T cells, herein referred to as AFF3-KO mice. Loss of *Aff3* expression in AFF3-KO mice T cells was confirmed using RT-qPCR (Figure 1C). The deletion of *Aff3* in T cells at the double-positive stage via expression of CD4-Cre does not disrupt their thymic development, as seen with deletion of other transcription factors such as TCF-1 (32) (Supplemental Figure 1 and Supplemental Figure 2; supplemental material available online with this article; https://doi.org/10.1172/jci.insight.207387DS1). CD4 and CD8 T cells were present in similar proportions in the thymus and spleen in WT (*Aff3*^wt/wt^ CD4-Cre^+^, WT) and AFF3-KO mice (Supplemental Figure 1). At steady state, AFF3-KO T cells remained phenotypically similar to WT cells (Supplemental Figure 1 and Supplemental Figure 2). Importantly, AFF3-KO CD8 T cells remained CD62L^+^ and CD44^lo^, establishing that AFF3 deficiency does not lead CD8 T cells to spontaneously acquire an antigen-experienced or activated phenotype (Supplemental Figure 2).

### AFF3 is necessary for CD8 T cells to accumulate in response to viral infection.

To determine if AFF3 plays a role in CD8 T cell responses to infection, AFF3-KO mice were crossed with P14 mice (TCR transgenic CD8 T cells specific to the LCMV GP_33-41_ epitope). We performed competitive coadoptive transfers in which 10,000 WT and AFF3-KO P14 CD8 T cells with disparate Thy expression (Thy1.1/1.1 or Thy1.1/1.2) were adoptively transferred at a 1:1 ratio to naive C57BL/6 mice (Thy1.2/1.2). This approach ensured that the adoptively transferred cells experienced the same inflammatory environment during priming and expansion. One day following coadoptive transfer, mice were infected with LCMV Armstrong (Arm, i.p. injection), which causes an acute infection (33–35).

On 5 (early expansion) and 8 (peak expansion) days postinfection (dpi) the frequency, number, and phenotype of the transferred cells was determined (Figure 1, D–G). We observed differences in the relative frequency and number of WT and AFF3-KO P14 CD8 T cells present in the spleen on 5 and 8 dpi (Figure 1D). Despite transferring an equal number of cells, AFF3-KO CD8 T cells were present at a higher frequency and number on 5 dpi (Figure 1, D and E). However, by 8 dpi, WT CD8 T cells were present at a higher frequency and number than the AFF3-KO CD8 T cells (Figure 1, D and F). This reduced accumulation of AFF3-KO P14 CD8 T cells was clearly reflected by the expansion ratio between 5 and 8 dpi. Compared with the number of cells on 5 dpi the WT CD8 T cells expanded on average 120 times by day 8; meanwhile, the AFF3-KO CD8 T cell population only expanded 70 times, on average (Figure 1G). This difference in expansion from 5 to 8 dpi results in nearly 8 × 10^6^ fewer P14 AFF3-KO CD8 T cells per spleen compared with WT CD8 T cells, suggesting AFF3 is required for optimal T cell accumulation. To address whether this represented a proliferation defect, we conducted ex vivo stimulation of WT and AFF3-KO CD8 T cells. P14 CD8 T cells were isolated from WT and AFF3-KO mice and activated ex vivo for 0, 1, 2, or 3 days using GP_33-41_ peptide, anti-CD28 antibody, and IL-2. AFF3-KO CD8 T cells proliferated more slowly than WT CD8 T cells, as demonstrated by the lower dilution of the cell proliferation dye, Tag-IT violet, suggesting that AFF3-KO CD8 T cells have a proliferative disadvantage compared with WT cells, leading to their reduced accumulation in response to infection (Figure 1H). Despite their defect in accumulation, both WT and AFF3-KO CD8 T cells were equally capable to produce the effector cytokines IFN-γ and TNF-α following restimulation (Supplemental Figure 3). This is in line with a previous study on the role in AFF3 in B cells, where they determined that AFF3 expression was not required for T cells to express effector cytokines (30). Together, these data indicate that *Aff3* expression in naive T cells is necessary for CD8 T cells to effectively proliferate and optimally accumulate during acute viral infection but is dispensable for cytokine production.

To dissect the mechanism regulating the accumulation defect observed for AFF3-KO CD8 T cells, we measured expression of important molecules regulating CD8 T cell proliferation and accumulation. CD25 is the high-affinity receptor for IL-2 and is important for maintained proliferation of CD8 T cells (36). AFF3-KO cells expressed less CD25 than WT cells at 5 dpi (Supplemental Figure 4A). By 8 dpi both WT and AFF3-KO T cells had very low expression of CD25, which is typical for T cells during acute infection (Supplemental Figure 4B). While there was a statistically significant difference between the WT and AFF3-KO CD8 T cells, it is unlikely to be of biological relevance due to the low expression of this receptor at this time point (Supplemental Figure 4B). To further investigate the regulation of expression of CD25 by AFF3 in CD8 T cells, we determined the expression of CD25 on CD8 T cells in vivo on days 1, 2, and 3 following infection, and we observed that AFF3-KO CD8 T cells expressed more CD25 on day 2 dpi, suggesting that they express this receptor more rapidly than WT CD8 T cells (Supplemental Figure 4C). This was confirmed via ex vivo activation of WT and AFF3-KO P14 CD8 T cells using GP_33-41_ peptide, anti-CD28, and IL-2. We observed no differences in CD25 expression on day 0, but AFF3-KO CD8 T cells had higher expression than WT CD8 T cells on days 1 and 2 of activation but not after 3 days of activation (Supplemental Figure 4D). Thus, AFF3-KO CD8 T cells express CD25 earlier than WT CD8 T cells but do not maintain its expression as long as WT CD8 T cells. Sustained expression of CD25 is key to maintain optimal CD8 T cell accumulation (36). Therefore, dysregulated timing of CD25 expression by AFF3-KO CD8 T cells may lead to their inability to optimally accumulate in response to infection.

AFF3-KO CD8 T cells may be receiving signals from IL-2 earlier in infection while lacking the continued signaling required for optimal CD8 T cell accumulation. To determine if changes in the timing of CD25 expression and signaling was responsible for the decreased accumulation of AFF3-KO CD8 T cells at 8 dpi, we took 2 approaches. We either blocked IL-2 using IL-2 antibodies or supplemented IL-2 with an IL-2/antibody complex (bypasses CD25 signaling) to increase IL-2 signaling in the recipient mice during infection, as previously described (36). On 8 dpi following treatment, we determined if accumulation of the AFF3-KO P14 CD8 T cells was restored (Supplemental Figure 4E). Neither blocking IL-2 by antibodies nor increasing IL-2 signaling via IL-2 antibody complex improved the accumulation of CD8 T cells (Supplemental Figure 4F). Therefore, while AFF3 deficiency changes the timing of CD25 expression by CD8 T cells, this change does not alter IL-2 responsiveness in a way that affects CD8 T cell accumulation. 

### AFF3 expression regulates the transcriptome of naive and activated CD8 T cells.

**Figure 2:**
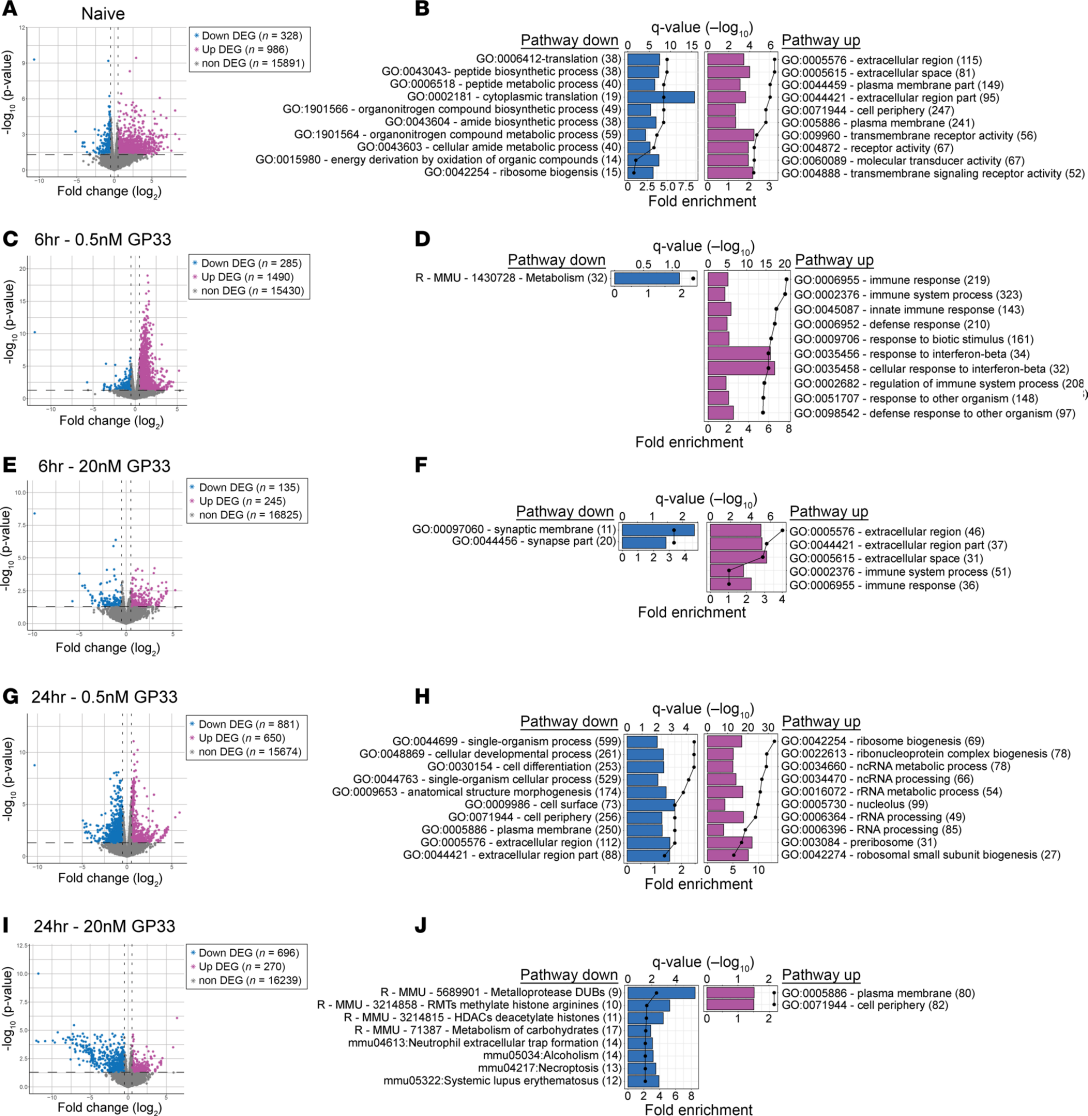
AFF3 deficiency changes the transcriptome of naive and activated CD8 T cells. RNA was isolated from splenic WT and AFF3-KO CD8 T cells from naive, 6-, and 24-hour activated cells with the indicated concentration of GP_33-41_ peptide, anti-CD28, and IL-2, before being processed for bulk RNA-seq. (**A**, **C**, **E**, **G**, and **I**) Volcano plot representing the DEGs in AFF3-KO CD8 T cells compared with WT CD8 T cells with the time point and treatment indicated. (**B**, **D**, **F**, **H**, and **J**) Bar chart showing the top changed pathways determined by *q* value for each time point and treatment. Bars indicate enrichment score for each pathway, and dots indicate *q* value. CD8 T cells were isolated from at least 3 mice in each condition and processed for sequencing.

Since differential expression of CD25 was not responsible for the reduced accumulation of AFF3-KO CD8 T cells and our phenotypic analysis did not reveal any other obvious changes that could explain the accumulation defect (Figure 1, D–G, and Supplemental Figure 2), we decided to take an unbiased approach to determine how AFF3 expression affects the transcriptome of CD8 T cells. We used bulk RNA-seq of naive WT and AFF3-KO CD8 T cells, and after T cell activation ex vivo to determine differences in the transcriptome. In AFF3-deficient naive CD8 T cells, there were 986 upregulated and 328 downregulated genes compared with WT CD8 T cells (Figure 2A). These differences suggest that AFF3 primarily acts as a negative regulator of transcription in naive cells, either directly or via other negative regulators. Pathway analysis demonstrated an increase in signaling receptor activity pathways but a decrease in metabolic, biosynthesis, and translation pathways (Figure 2B). When WT and AFF3-KO CD8 T cells were activated with 0.5 nM GP_33-41_ peptide, anti-CD28 antibody, and IL-2 for 6 hours, there were 1,490 upregulated and 285 downregulated genes in the AFF3-KO CD8 T cells, again highlighting the role of AFF3 as a negative regulator of transcription early during T cell activation (Figure 2C). Pathway analysis denoted an increase in immune response and recognition of a pathogen pathway with a continued decrease in metabolic pathways, which are required for the generation of building blocks that support sustained proliferation (37) (Figure 2D). At 6 hours of activation with 20 nM GP_33-41_ peptide, anti-CD28, and IL-2, AFF3-KO CD8 T cells had 245 upregulated and 135 downregulated genes (Figure 2, E and F). This different number of genes regulated when cells are stimulated with higher concentration of peptide suggests that AFF3 may have a differential role depending on the strength of TCR stimulation. This is potentially linked to the fact that TCR simulation can partially downregulate *Aff3* expression and stronger stimulation may lead to a more profound loss of *Aff3*, leading to fewer differently expressed genes (DEGs) being observed with AFF3-KO CD8 T cells. After 24 hours of activation with 0.5 nM GP_33-41_ peptide, AFF3-KO CD8 T cells had 650 upregulated and 881 downregulated genes (Figure 2G). Pathway analysis showed an increase in RNA processing and ribosome biogenesis pathways with a decrease in cell differentiation and developmental processes pathways (Figure 2H). Finally, after 24 hours of activation in 20 nM GP_33-41_, AFF3-KO CD8 T cells had 270 upregulated and 696 downregulated genes (Figure 2I). Pathway analysis showed that there was a decrease in metabolism of carbohydrates and metalloprotease deubiquitinase enzymes (DUBs) (Figure 2J). DUBs are important during the T cell response because they regulate the signaling downstream of TCR such as NFAT signaling and NF-κB signaling (38). Interestingly, after 24 hours of stimulation, we observed more genes downregulated in AFF3-KO CD8 T cells compared with WT CD8 T cells, suggesting that deletion of AFF3 may negatively affect the expression of genes that are required as the cells become activated and begin differentiating. Together, these data establish that AFF3 plays an important role in regulating cell signaling and metabolic pathways. Interestingly, the data highlight that AFF3 differentially regulates the transcriptome of CD8 T cells depending on the concentration of antigen used for stimulation and the duration of stimulation.

One notable transcriptional change at 24 hours of activation was the increase in the gene expression of *Fcgr2b* and *Fcgr3a* (Supplemental Figure 5A). *Fcgr2b* has been previously described to prevent T cell proliferation (39). To confirm that these receptors are upregulated at the protein level, we evaluated their expression using flow cytometry during LCMV infection. We confirmed that expression of CD32 (FCGR2B) but not CD16 (FCGR3A) was increased on AFF3-KO CD8 T cells on 1 and 2 dpi compared with WT CD8 T cells (Supplemental Figure 5, B and C). Next, we asked if blockade of CD32/CD16 would restore the accumulation of the AFF3-KO CD8 T cells. Despite the increased expression of CD32, we found that CD32/CD16 blockade was not sufficient to restore CD8 T cell accumulation (Supplemental Figure 5, D and F). Thus, while AFF3 downregulates the expression of CD32 early in infection, which may play a role in T cell responses at later time points, this change does not regulate the expansion of CD8 T cells.

### AFF3 is needed to maintain a quiescent metabolism.

**Figure 3:**
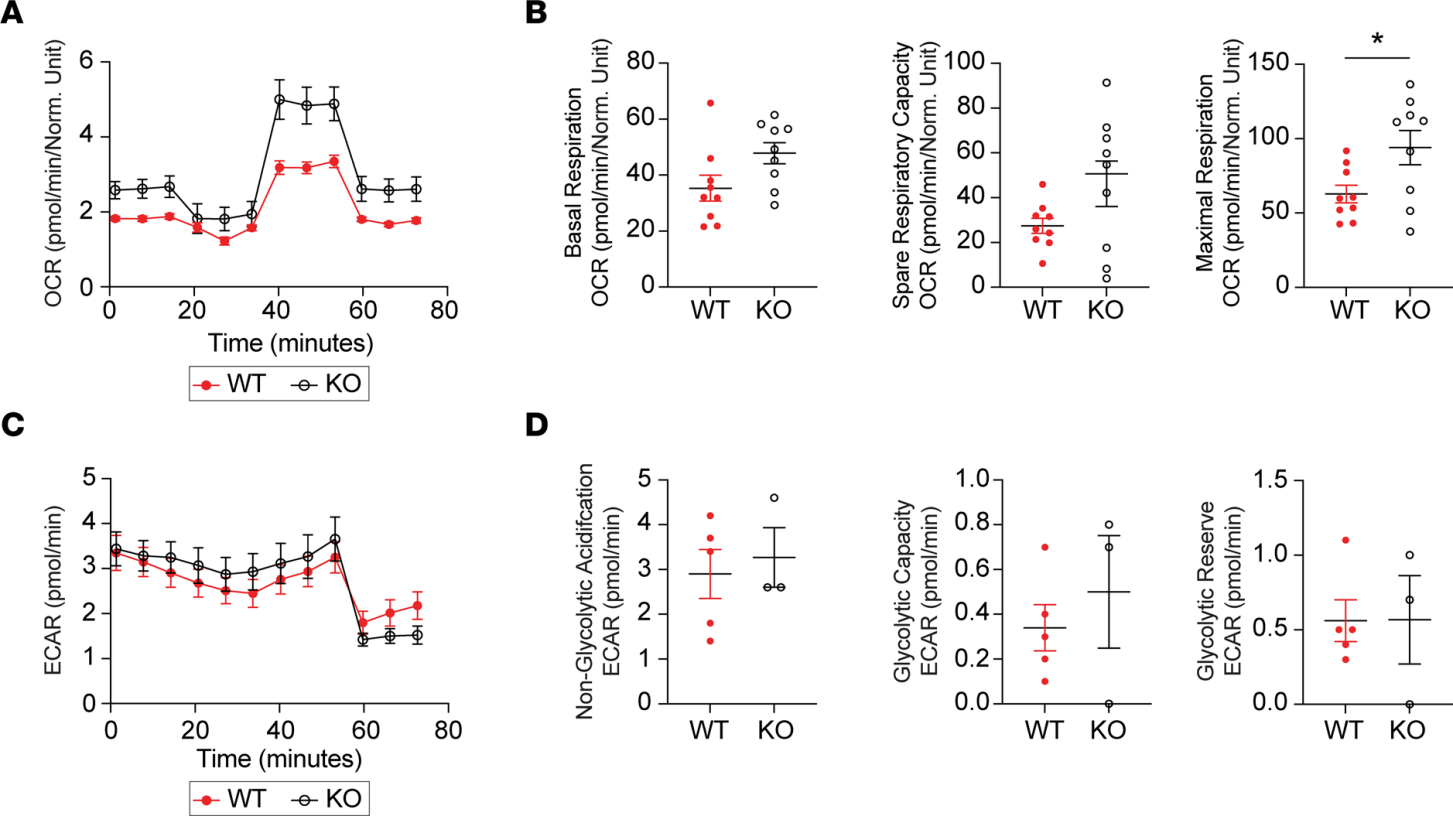
AFF3 is necessary to maintain CD8 T cell metabolic quiescence CD8 T cells. Splenic naive WT and AFF3-KO CD8 T cells were isolated, and metabolism was analyzed using a Seahorse XF analyzer. (**A**) OCR overindicated time using mitochondrial stress test kit. (**B**) Basal respiration (first panel), spare respiratory capacity (middle panel), and maximal respiration (last panel) were determined by the OCR in the Seahorse analysis. (**C**) ECAR over time using the glycolysis stress test. (**D**) Nonglycolytic acidification (first panel), glycolytic capacity (middle panel), and glycolytic reserved (last panel) determined by the ECAR of Seahorse analysis. (**A**–**D**) Representative of 2 experiments with *n* = 3 mice per group with 3 technical replicates per mouse. (**B** and **D**) Groups compared using unpaired Student’s *t* test, *P <0.05. Data represent mean ± SEM.

Since one of the major changes observed via transcriptional analysis of naive and recently activated CD8 T cells was metabolism pathways, we next profiled the cellular metabolism of WT and AFF3-KO CD8 T cells using Seahorse real-time metabolic analysis. Interestingly, naive AFF3-KO CD8 T cells trended to have higher basal respiration and spare respiratory capacity (SRC) and had significantly higher maximal respiration compared with WT CD8 T cells as determined by mitochondrial stress test (Figure 3, A and B). Naive CD8 T cells use low levels of oxidative phosphorylation (OXPHOS), activated CD8 T cells rely on glycolysis and memory, and virtual memory CD8 T cells use OXPHOS but have a higher SRC and maximal respiration compared with naive cells (40, 41). This increased SRC allows for memory cells to respond more quickly to antigen by rapidly increasing energy generation (41). Therefore, naive AFF3-KO CD8 T cell metabolism was more reminiscent of memory CD8 T cells rather than what is typically observed for naive CD8 T cells (40, 41). This is particularly interesting, as naive AFF3-KO CD8 T cells adopt this change in metabolism without acquiring an antigen-experienced phenotype, suggesting that AFF3 downregulates OXPHOS in CD8 T cells independently of antigen encounter. Additionally, we compared the glycolytic capacity of naive WT and AFF3-KO CD8 T cells, and we found that there were no differences present with neither WT nor AFF3-KO CD8 T cells adopting a glycolytic metabolism, which is to be expected for naive cells (Figure 3, C and D). This suggests that, while metabolic quiescence is disrupted in naive AFF3-KO CD8 T cells, they do not spontaneously switch to a glycolytic metabolism. We determined if the changes in the metabolism in AFF3-KO CD8 T cells were due to changes in mitochondrial health. We tested total cell reactive oxygen species, mitochondrial membrane potential, mitochondrial superoxide levels, and mitochondrial mass. We observed no differences with any of these markers of mitochondrial health between WT and AFF3-KO CD8 T cells (Supplemental Figure 6, A–D). These data suggest that AFF3 regulates T cell metabolism independently of any obvious defect in mitochondria. Together, these data support that AFF3 expression is necessary in naive CD8 T cells to maintain a quiescent metabolism even prior to antigenic encounter.

### AFF3 deficiency changes the expression of key solute carriers and leads to a reduction in glucose metabolism.

**Figure 4:**
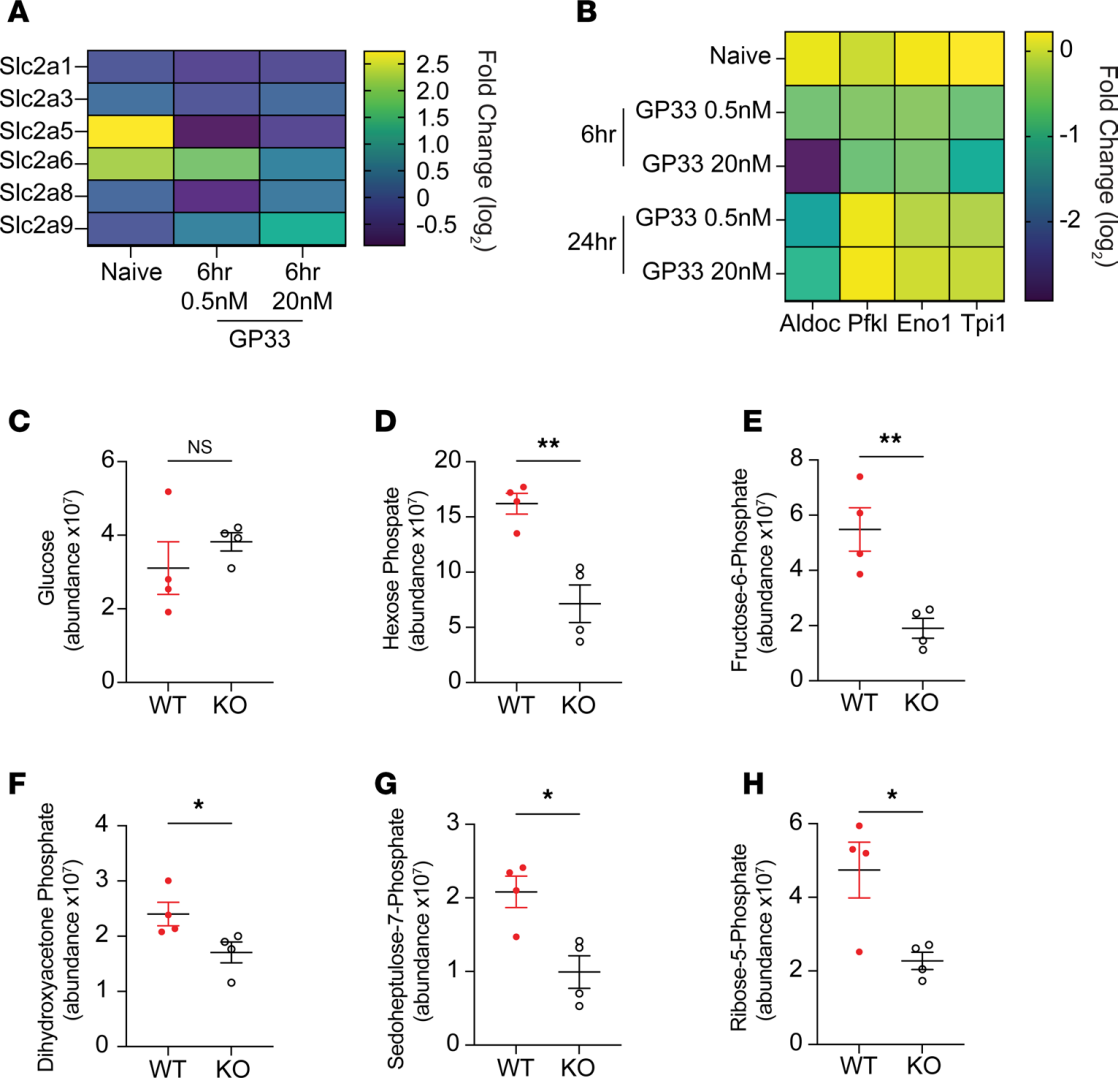
AFF3 deficiency changes the expression of solute carriers and results in decreased glycolysis following activation. (**A**) Top significantly changed solute carrier genes in naive splenic WT and AFF3-KO CD8 T cells determined by RNA-seq analysis. (**B**) Heatmap of significantly changed genes in the glycolysis pathway determined by RNA-seq analysis. (**C**–**H**) Abundance of the indicated glycolysis or PPP intermediate determined by metabolomics analysis on splenic WT or AFF3-KO CD8 T cells activated ex vivo for 24 hours with anti-CD3 and anti-CD28. (**A** and **B**) At least 3 mice per group per conditions. (**C**–**H**) 4 mice per group and groups analyzed with unpaired Student’s *t* test, *P < 0.05, **P < 0.01. Data represent mean ± SEM.

Since AFF3 was shown to regulate naive CD8 T cells metabolism, we next analyzed our RNA-seq dataset for potential regulators of metabolism that might be dysregulated in AFF3-KO CD8 T cells. One important regulator of metabolic activity is the expression of solute carriers that regulate the availability of key metabolite to fuel cell activation and proliferation (42–44). Our RNA-seq data establish that AFF3-KO CD8 T cells increase the expression *Slc2a5*, known as GLUT5, which is the selective receptor for fructose transport (45) (Figure 4A). In general, CD8 T cells do not express GLUT5 but rather express GLUT1 or GLUT3 (46, 47). At the same time, naive and activated AFF3-KO CD8 T cells have lower expression of *Slc2a1*, known as GLUT1, the primary glucose transporter for T cells (Figure 4A). We also observed that AFF3 deficiency resulted in decreased expression of several glycolytic enzymes following activation (Figure 4B). Together, these data suggest that AFF3-KO CD8 T cells may be unable to efficiently metabolize glucose to fuel their proliferation.

**Figure 5:**
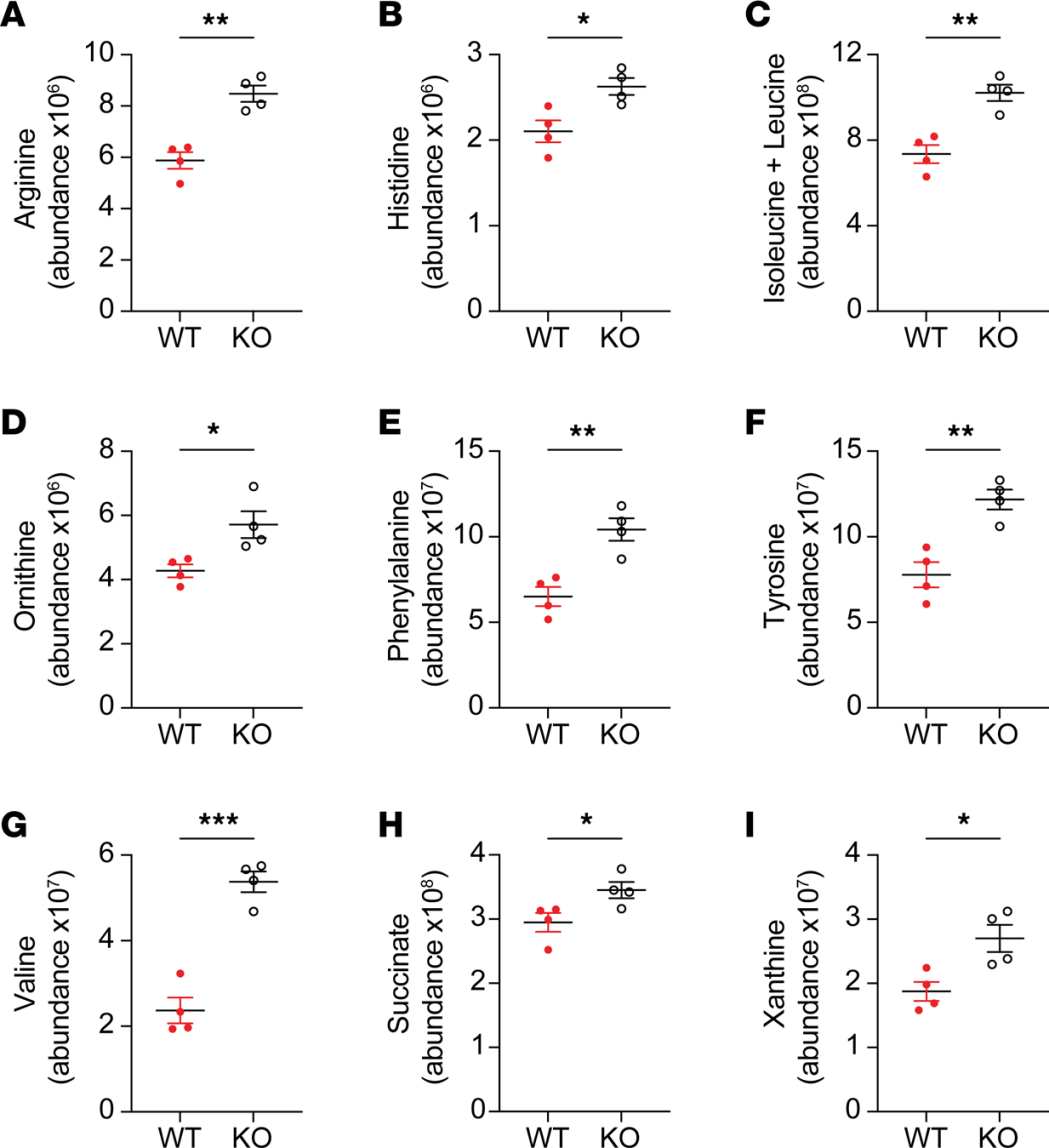
AFF3 deficiency leads to the accumulation of amino acids and other metabolic intermediates following CD8 T cell activation. Splenic WT or AFF3-KO CD8 T cells were isolated and activated ex vivo for 24 hours with anti-CD3 and anti-CD28 and metabolite abundance measured via metabolomics analysis. (**A**–**I**) Abundance of the indicated metabolite in WT or AFF3-KO CD8 T cells. Four mice per group were used, and groups were analyzed with unpaired Student’s *t* test. *P < 0.05, **P < 0.01, ***P < 0.001. Data represent mean ± SEM.

To determine if the switch to glycolysis was affected in AFF3-KO CD8 T cells, we performed metabolomics on WT and AFF3-KO CD8 T cells following anti-CD3 and anti-CD28 activation for 24 hours. Metabolomics profile revealed several changes between WT and AFF3-KO CD8 T cells. While glucose levels were not significantly changed, we observed that several key intermediates in both the glycolysis and the pentose phosphate pathways (PPP) were reduced in AFF3-KO CD8 T cells (Figure 4, C–H). This suggests that they are poorly metabolizing glucose and not switching to glycolysis following activation. In addition, we observed that several amino acids, including the branched amino acids (BCAA) valine, leucine and isoleucine, were upregulated in AFF3-KO CD8 T cells (Figure 5, A–G). This was accompanied by increased succinate and xanthine in AFF3-KO CD8 T cells (Figure 5, H and I). This metabolic profile with reduced glycolysis and PPP intermediates and increased amino acids is reminiscent of cells with disrupted metabolism such as senescent or aging T cells and likely explains the diminished capacity of AFF3-KO CD8 T cells to accumulate following infection (48, 49).

### AFF3 is involved in preventing premature immune aging.

**Figure 6:**
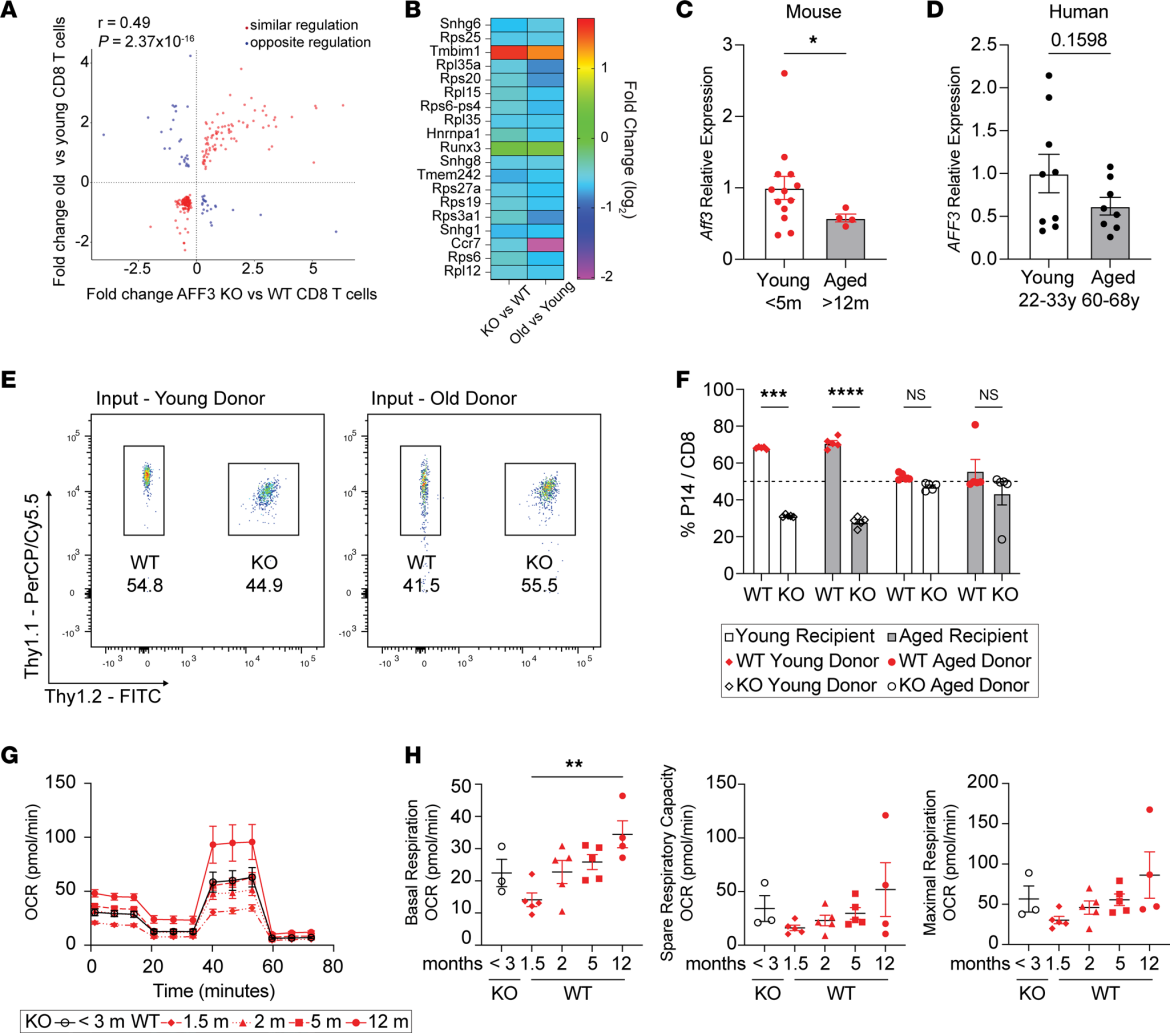
AFF3 expression is decreased with age and is associated with a loss of metabolic quiescence. (**A**) Graph showing the correlation of DEGs in naive AFF3-KO CD8 T cells versus WT CD8 T cells and aged versus young WT CD8 T cells. (**B**) Heatmap showing top changed genes in AFF3-KO CD8 T cells and aged WT CD8 T cells compared with young WT controls. (**C**) Relative gene expression of *Aff3* in young (<5 months) and aging (>12 months) splenic WT CD8 T cells determined by RT-qPCR analysis. (**D**) Relative *AFF3* expression in young (22–33 years old) versus aged human (60–68 years old) naive CD44^lo^ CD8 T cells isolated from PBMCs determined by RT-qPCR analysis. (**E**) Representative flow plots showing the frequency of the cotransfer input of aging CD8 T cells donors or young CD8 T cell donors. (**F**) Frequency of transferred CD8 T cells in the spleens of young or aged WT recipient mice 8 dpi with LCMV Arm. (**G**) Metabolic analysis of splenic CD8 T cells. OCR over time using a mitochondrial stress test. (**H**) Basal respiration (first panel), spare respiratory capacity (middle panel), and maximal respiration (last panel) were determined by the OCR in the Seahorse analysis. (**C**, **E**, and **F**) Representative of 2 experiments, *n* = 3–5 per group. (**D**) Representative of 1 experiment, *n* = 8–9 samples per group. (**G** and **H**) Representative of 1 experiment, *n* = 3–4 per group with 3 technical replicates per mouse, age of mice in each sample indicated in the figure legend. (**C**) Groups compared using unpaired Student’s *t* test, *P <0.05. (**F**) Groups compared using paired Student’s *t* test, ***P < 0.001, ****P < 0.0001. (**H**) Groups compared using 1-way ANOVA with post hoc test, **P < 0.01. Data represent mean ± SEM.

The differences we observed for AFF3-KO CD8 T cells, such as decreased metabolic quiescence, decreased proliferation, and an altered metabolic profile, are reminiscent of the loss of quiescence that occurs in T cells in aged individuals (5, 49–54). Therefore, we determined if AFF3 is important to prevent immune aging. To start addressing this, we compared the DEGs from our RNA-seq data to published data that compare the DEGs of young and aged CD8 T cells (55). We found that the DEGs changes in the AFF3-KO CD8 T cells were significantly correlated with changes (both upregulated and downregulated genes) from aged CD8 T cells (Figure 6, A and B). This suggests that AFF3-KO CD8 T cells are more transcriptionally similar to aged WT CD8 T cells than their age-matched WT CD8 T cells counterparts. Interestingly, this comparison holds true, even though naive AFF3-KO CD8 T cells do not develop a memory-like phenotype as opposed to what is likely occurring among tissue dwelling CD8 T cells in aging mice. This suggests that AFF3 prevents the development of immune aging independently of T cell activation or acquisition of a memory-like phenotype. To further address if loss of AFF3 is associated with aging in T cells, we compared gene expression of *Aff3* in young and aging mice. We found that aging mice (>12 months) had lower expression of *Aff3* compared with young WT mice (<5 months), demonstrating that *Aff3* declines with age in CD8 T cells (Figure 6C). Since we observed that the AFF3-KO CD8 T cells from the mice share transcriptomic similarities to aged WT CD8 T cells and that *Aff3* expression decreases with age in murine T cells, we next asked if this was also observed in naive human CD8 T cells. We tested human PBMC samples from young (22–33 years old) and aged (60–68 years old) donors with equal representation of male and female patients (Supplemental Table 1). We isolated naive CD8 T cells and compared *Aff3* expression. We enriched for naive cells instead of total CD8 T cells to control for the fact that humans, as opposed to mice maintained in specific pathogen–free conditions, will accumulate antigen-experienced memory CD8 T cells from their history of infection, and we wanted to compare *Aff3* expression in foreign-antigen inexperienced CD8 T cells (53, 56, 57). While we found more variability among patients, we observed a trend for decreased expression of *Aff3* in naive CD8 T cells with age (Figure 6D). These data further suggest that the AFF3-deficient CD8 T cells are more similar to aged CD8 T cells and that AFF3 may play a role in restraining immune aging in CD8 T cells in both humans and mice.

To address whether reduced *Aff3* expression in aged mice yields similar accumulation defects to what we observed for young AFF3-KO CD8 T cells, we compared the CD8 T cell response to viral infection in AFF3 KO and WT CD8 T cells from aging donor mice greater than 6 months of age. In line with the reduction of *Aff3* expression with age, the proliferation advantage of the WT P14 CD8 T cells over the AFF3-KO P14 CD8 T cells was lost when aging donors were used (Figure 6, E and G). This suggests that decreased *Aff3* gene expression that occurs naturally in CD8 T cells during aging leads them to respond more similarly to AFF3-KO cells than young WT CD8 T cells that express high levels of *Aff3*. To determine if this was strictly CD8 T cell intrinsic or if the aged environment also played a role in reducing proliferation and accumulation, we cotransferred AFF3-KO and WT CD8 T cells that were either from young donors (6–12 weeks) or aging donors (>6 months) into naive young (6–8 weeks) and older (>12 months) recipient mice. We found that, with young donor cells, AFF3-KO P14 CD8 T cells accumulated less than the WT donor cells at 8 dpi independently of the age of the recipient mice (Figure 6, E and F). Conversely, the aged donor cells expanded equally at 8 dpi, regardless of their genotype, independently of the age of the recipients (Figure 6, E and F). This suggests that the loss of *Aff3* leads to T cell intrinsic immune aging and that the natural loss of *Aff3* that occurs in WT mice during aging is a component of the loss of T cell responsiveness in aged individuals, which is independent of other environmental factors in aged mice in the short time frame of our experiments.

Finally, we asked if the changes in the metabolism of aged CD8 T cells were the similar to those observed in young AFF3-KO CD8 T cells via a mitochondrial stress test of WT CD8 T cells from mice of various ages. We observed that WT CD8 T cells became progressively less metabolically quiescent with age, as noted by an increase in OXPHOS activity (Figure 6, G and H). Furthermore, we found that KO CD8 T cells from mice younger than 3-month-old had a similar metabolism to that of WT CD8 T cells from 5-month-old mice (Figure 6, G and H). Conversely, WT CD8 T cells from younger mice were more quiescent metabolically than similarly aged AFF3-KO CD8 T cells (Figure 6, G and H). This suggests that genetic loss of AFF3 accelerates age-associated loss of metabolic quiescence but also suggests that factors beyond loss of AFF3 play a role in the loss of metabolic quiescence. Therefore, our data support a role for AFF3 in maintaining T cell quiescence and responsiveness and suggest that age-related loss of *Aff3* expression in CD8 T cells plays a role in their progressive dysfunction.

## Discussion

Active maintenance of naive CD8 T cell quiescence is critical to their ability to respond to infection while preventing inappropriate activation and immune pathology. Changes to the naive state of CD8 T cells, such as those observed in immune aging, are associated with T cells becoming less responsive and less protective against viral infection (50–53, 56, 58). Similarly, loss of quiescence downstream of mTORC1 activation in mice lacking TSC1 is associated with spontaneous activation and poor responsiveness to infection (7, 9). Therefore, quiescence must be tightly regulated to ensure optimal T cell responses. Dissecting the mechanisms that keep naive cells in a quiescent state is important to understanding how to improve and regulate immune responses and potentially restore T cell function in aged individuals. Herein, we found that the transcription factor AFF3 is a regulator of naive CD8 T cell metabolic quiescence and *Aff3* expression in naive CD8 T cells is required for their capacity to accumulate robustly when activated. This is correlated with a reduced capacity to engage glycolysis following activation, despite higher resting mitochondrial metabolism. Furthermore, we observed that *Aff3* expression is reduced with age in both in murine and human CD8 T cells, suggesting that AFF3 is involved in preventing immune aging by regulating metabolic quiescence. Thus, AFF3 may be a therapeutic target to improve the response of aged human T cells to vaccination and disease. Further studies are needed to understand the mechanisms that decrease *Aff3* expression with age and how they can be targeted to reinvigorate the T cell response.

In this study, we identified that naive AFF3-KO CD8 T cells exhibit dysregulated mitochondrial metabolism with higher maximal respiration and a trend toward higher SRC, which resembles the metabolic state of memory T cells (40, 41). In memory CD8 T cells, these metabolic changes allow the cells to respond more quickly to antigen (41, 59), which may explain why the AFF3-KO CD8 T cells activate faster during ex vivo stimulation. However, memory cells switch to glycolysis upon activation, and our RNA-seq data identified that AFF3-KO CD8 T cells have decreased expression of glycolysis enzymes after activation. Furthermore, metabolomics analysis demonstrated a reduction in glycolysis and PPP intermediates in AFF3-KO CD8 T cells following activation. This suggests that the AFF3-KO CD8 T cells do not switch to glycolysis as effectively as WT CD8 T cells. Thus, our data suggest that activated AFF3-KO CD8 T cells continue to rely on mitochondrial respiration even after activation, which is not typically observed for effector CD8 T cells (13, 18, 37, 60). It is possible that the delay in the switching to glycolysis inhibits the ability of the AFF3-KO CD8 T cells to continue to accumulate throughout infection. This is supported by a decrease in glycolytic enzymes expressed by activated AFF3-KO CD8 T cells and their reduced production of glycolysis and PPP intermediates. Interestingly, we observed an increase in amino acids, including BCAA. Increased presence of BCAAs has been associated with T cell senescence, and increased amino acids have also been observed in aging T cells and linked to their defective proliferation (48, 49).

The change in metabolic capacity of AFF3-KO CD8 T cells may be linked to their differential expression of solute carriers and their capacity to incorporate glucose (42–44). Naive AFF3-KO CD8 T cells have increased expression of GLUT5 and decreased expression of GLUT1. However, metabolomics did not indicate a reduction in glucose in AFF3-KO CD8 T cells, so it remains unclear if glucose uptake is affected. It is possible that WT CD8 T cells uptake more glucose than AFF3-KO CD8 T cells but that this glucose is efficiently used for glycolysis, thereby reducing the levels that were measured. This is supported by the reduced glycolysis and PPP intermediates observed in AFF3-KO CD8 T cells, suggesting poor glucose utilization. This is further supported by the more profound proliferation defect observed for AFF3-KO CD8 T cells ex vivo, where glucose is the primary energy source. This contrasts with what we observed in vivo, where AFF3-KO CD8 T cells can maintain proliferation for the first 5 days before being outpaced by WT CD8 T cells, potentially via their use of other energy sources. In addition, our data indicate that AFF3-KO CD8 T cell may be using fructose as an energy source compared with glucose by upregulating expression of GLUT5. Since fructose enters the glycolysis cycle later than glucose, it may be contributing to the less robust accumulation of cells by bypassing the PPP (61). In addition, fructose can lead to an increase in glucose uptake by hepatic cells (62), suggesting that it may help AFF3-KO CD8 T cells acquire nutrients. However, since glucose is a better fuel for CD8 T cell proliferation, this fructose dependence, even if it allows eventual glucose uptake, combined with their reduced capacity to engage glycolysis, likely poses a substantial disadvantage for AFF3-KO CD8 T cells. This disadvantage is likely exacerbated in the context of our experiments where cells are cotransferred and, therefore, competing for nutrients. It remains unclear why AFF3 restrains the expression of GLUT5, which is not typically expressed by CD8 T cells, and whether this is a direct suppression or whether increased expression of GLUT5 is a consequence of changing expression of other solute carrier, such as the decrease in expression of GLUT1. The regulation of GLUT5 and the role of fructose in regulating the metabolism of AFF3-KO CD8 T cells will be the subject of future investigations.

Interestingly, we observed that naive AFF3-KO CD8 T cells had a metabolic profile reminiscent of memory or virtual memory T cells. However, this occurs in AFF3-KO CD8 T cells without the acquisition of an antigen-experienced phenotype such as high expression of CD44. Even virtual memory CD8 T cells, which are considered to be antigen inexperienced since they have not encountered foreign antigens, express higher levels of CD44 likely due to their interaction with self-antigens during homeostatic proliferation (63, 64). To our knowledge, this is the first description of a loss of metabolic quiescence in CD8 T cells independently of TCR signaling or changes in activation marker expression. Our data suggest that naive cells are poised for metabolic changes toward increased OXPHOS but that this pathway is actively suppressed by AFF3 independently of antigen encounter. Interestingly, as opposed to virtual memory T cells, AFF3-KO CD8 T cells accumulate less than WT CD8 T cells to antigenic activation. These data suggest that metabolic changes in the absence of other reprogramming induced by TCR signaling are detrimental to CD8 T cell function. These questions will be the subject of future investigation.

Additionally, our data establish that AFF3 deficiency leads to increased expression of the inhibitory receptor CD32 on CD8 T cells responding to infection. While our data support that this change is not responsible for the decreased accumulation of CD8 T cells during infection, the increase in CD32 expression may have important consequences for the responsiveness of memory CD8 T cells to reinfection. CD32 has been shown to mitigate the secondary response of CD8 T cells in the presence of antibodies (65), and the increased expression of CD32 by AFF3-KO CD8 T cells suggests that this could lead to a reduction in the functionality of memory CD8 T cells. It is currently unclear if AFF3 expression is important for memory CD8 T cell function, but the ImmGen database indicates that AFF3 expression is increased in memory CD8 T cells after being downregulated in effector CD8 T cells, suggesting that it may also play a role in their function and quiescence; this will be the subject of future investigations (66).

Finally, our data support that AFF3 expression is required to prevent premature T cell aging. Specifically, AFF3 regulates metabolic quiescence, and this is progressively lost as T cells age and reduce their expression of *Aff3*. In addition, AFF3-KO CD8 T cells share a metabolomic profile, including decreased glycolysis and PPP intermediates and increased amino acids, with the metabolomic profile of aged CD4 T cells (49). While we observed that AFF3 is unlikely to be the only regulator of metabolic quiescence that is lost during aging, as mouse T cells became more metabolically dysregulated than AFF3-KO CD8 T cells when they were aged longer, our data support that AFF3 is a potential target to reinvigorate T cell function in both mice and humans. It is currently unclear what signals drive the loss of *Aff3* with aging, but our data in human samples suggest that this loss occurs independently of antigen encounter since it was observed in naive cells. Since our data establish that inflammatory cytokines reduce expression of *Aff3* in CD8 T cells, it is likely that the dysregulated cytokine milieu observed in aging may lead to reduced *Aff3* expression.

Together, our work has established that AFF3 plays an important role as a regulator of metabolic quiescence in CD8 T cells. In its absence, CD8 T cells become metabolically dysregulated and accumulate poorly in response to viral infection. Importantly, *Aff3* expression is progressively lost in aging, and AFF3-KO CD8 T cells adopt a transcriptome and metabolic differences that are reminiscent of aged T cells.

## Methods

### Sex as a biological variable.

Both males and females were used for mouse and human studies.

### Mice.

*Aff3*^fl/fl^ mice were made by obtaining sperm from the European Mutant Mouse Archive (EMMA). Initial in vitro fertilization was performed at McGill University. FRT cassette in the EMMA construct was removed by crossing *Aff3*^fl/fl^ mice with a flipase expressing mouse from Jackson Laboratories (strain no. 009086). Progeny mice were crossed with CD4-Cre mice purchased from Jackson Laboratories (strain no. 017336). WT P14 TCR–transgenic mice (LCMV–derived GP_33–41_ epitope) (67) were bred at IUSM. *Aff3*^fl/wt^ CD4-Cre^+^ mice were bred with P14 mice to generate AFF3-KO P14 mice. Mice were bred as *Aff3*^fl/wt^ heterozygous CD4-Cre^+^ to obtain both WT and KO littermates. *Aff3* genotype was confirmed by PCR. TCR transgenic and congenic marker status were confirmed by flow cytometry. All WT mice in the study were CD4-Cre^+^ to control for any potential Cre effects.

### Adoptive transfer.

One day before infection, 10,000 AFF3-KO and WT P14 CD8 T cells were cotransferred at a 1:1 ratio into naive C57BL/6 mice via i.v. injection. Donor cells were isolated from the spleen of WT and KO TCR-transgenic mice. Donor cells were confirmed to be naive (CD62L^+^CD44^lo^) and expressing the correct Thy congenic marker by flow cytometry. After cells were mixed, the input ratio was confirmed using flow cytometry prior to injection.

### Ex vivo culture of T cells.

CD8 T cells were enriched using the EasySep CD8 enrichment kit (StemCell Technologies, 19853). Cells were counted and 4.5 × 10^5^ cells were added to each well of a 96-well plate. Cells were activated with 2 μg/mL of plate-bound anti-CD3 and 2 μg/mL of anti-CD28 or 2 μg/mL of anti-CD28 and either 0.05 nM GP_33-41_ peptide or 20 nM GP_33-41_ peptide (Bio-Synthesis). Cell cultures were supplemented with 10 ng/mL recombinant mouse or human IL-2 (Peprotech, 212-12-20UG or 212-12-50UG).

### Virus.

LCMV Arm was provided by J. Harty (The University of Iowa, Iowa City, IA) and propagated as previously described (29, 68, 69). Viral titers were determined as previously described (70–72). Mice were infected with 2 × 10^5^ plaque forming units (PFU) of LCMV Arm in 200 μL of PBS via i.p. injection.

### Flow cytometry.

Single-cell suspensions were generated from spleens at the indicated time points in RP5 media (RPMI-1640 (Thermo Fisher Scientific, SH30255FS) + SC (HEPES, L-glutamine, pen/strep, gentamicin, B-ME) + 5% FBS (R&D Systems Inc., S11550). Organs were mechanically disrupted and filtered through a 70 μM conical filter. Cells were treated with 1x RBC lysis (BioLegend, 420302) for 5 minutes before being washed and resuspended in RP5. Cells were counted in 0.1% Trypan blue in PBS (VWR, 470302-020), Trypan Blue 0.4% (Fisher Scientific, 15250061), 10% Sodium Azide (Fisher Scientific, NC9720916) on a hemocytometer. For staining, cells were added to a 96 well plate and washed with FACS buffer (1x PBS, 1% FBS, 0.2% Sodium Azide) and pelleted. Cells were resuspended with 100 μL FACS buffer containing antibody cocktail and incubated at 4°C for 20 minutes, followed by another wash. Cells were treated with fixation buffer (BioLegend, 420801) and incubated at 4°C for 10 minutes, followed by a wash. Cells were resuspended in FACS buffer and analyzed on a Fortessa flow cytometer. Data was analyzed using the latest version of FlowJo (BD Biosciences, Version 10.10.0).

For intracellular flow cytometric analysis, surface staining was performed as described above. Fixed cells were washed with 1x permeabilization buffer (BioLegend, 421002) and stained with intracellular antibody diluted in 1x permeabilization buffer and incubated at 4°C for 20 minutes. Cells were washed with 1x permeabilization buffer and resuspended in FACS buffer then analyzed as described above.

For nuclear flow cytometric analysis, surface staining was performed as described above. The cells were fixed using a 1:4 dilution of FOXP3 fix (Thermo Fisher Scientific, 50-112-9081 and 50-112-9082) for 30 minutes at 4°C and cells washed with 1x permeabilization buffer. Cells were stained with nuclear antibody diluted in 1x permeabilization buffer and incubated at 4°C for 20 minutes. Cells were washed with 1x permeabilization buffer and resuspended in FACS buffer and analyzed as described above.

### Antibodies.

The antibodies used in this study were as follows in various combination of fluorophores: CD8 (53-6.7, BioLegend), Vα2 (B20.1, BioLegend), Thy1.1 (OX-7, BioLegend), Thy1.2 (53-2.1, BioLegend), CD44 (IM7, BioLegend), CD62L (MEL-14, BioLegend), CD25 (3C7, BioLegend), CD69 (H1.2F3, BioLegend), CD4 (GK1.5, BioLegend), CD3ε (145-2C11, BioLegend), CD127 (A7R34, BioLegend), CD122 (5H4, BioLegend), CD132 (TUGm2, BioLegend), EOMES (W17001A, BioLegend), Ly6C (HK1.4, BioLegend), CD215 (DNT15Ra, Thermo Fisher Scientific), CD27 (LG.3A10, BioLegend), CD43 (1B11, BioLegend), CD43 (S11, BioLegend), CD43 (S7, BD Biosciences), Ki-67 (B56, BD Biosciences), CD32 (S17012B, BioLegend), CD16 (S17014E, BioLegend), TCF-1 (S33-966, BD Biosciences), GranzymeB (GB11, BioLegend), Perforin (S16009A, BioLegend), GranzymeA (3G8.5, BioLegend), cMYC (SH1-26E7.1.3, Miltenyi Biotec), CellROX Green (C10444, Thermo Fisher Scientific), JC-1 Dye (T3168, Thermo Fisher Scientific), MitoSox (M36008, Thermo Fisher Scientific), Mitospy Green FM (424805, BioLegend), 2-NBDG (N13195, Thermo Fisher Scientific), purified CD3 (145-2C11, BioLegend), and purified CD28 (37.51, BioLegend).

### qPCR.

CD8 T cells were enriched using the EasySep CD8 enrichment kit (StemCell Technologies; mouse, 19853; Human, 17968). RNA was extracted using a QIAGEN RNeasy mini kit (QIAGEN, 74104). RNA concentration and quality was quantified using a NanoDrop 2000c Spectrophotometer. RNA was converted to cDNA using the iScript cDNA Synthesis Kit (BioRad, 1725038). Gene expression was quantified using PowerTrack SYBR Green Master Mix for qPCR kit (Thermo Fisher Scientific, A46109) using conditions defined by the manufacturer using a Quanstudio 6flex instrument (Thermo Fisher Scientific, 4485689). Expression was normalized to TATA binding protein as an internal control and depicted as a relative fold change using the ΔΔCt method, compared with the mean of the control group.

Primers used for qPCR were as follows: mouse *Aff3*-forward (TCGAGTCGCTGTGTGTCTATG), mouse *Aff3*-reverse (GGTTGGAGAGTTCATCCCCC), mouse *Tbp*-forward (TGGAATTGTACCGCAGCTTCA), mouse *Tbp*-reverse (ACTGCAGCAAATGCGTTGGG), human *AFF3*-forward (GCCCTACAAGACTAACAAGGGG), human *AFF3*-reverse (ACTCCAACGAGATGACTCTGAT), human *TBP*-forward (CACGAACCACGGCACTGATT), and human *TBP*-reverse (TTTTCTTGCTGCCAGTCTGGA).

### Seahorse real-time metabolic analysis.

OCR and ECAR were determined on a Seahorse XF Pro Analyzer using the mitochondrial (Agilent, 103015-100) or glycolysis stress test kits (Agilent, 103020-100) per manufacturer’s protocol. Briefly, CD8 T cells were isolated and enriched using the EasySep CD8 enrichment kit (StemCell Technologies, 19853). In total, 2 × 10^5^ cells were added to a 96-well plate coated with 5.2 μg/cm^2^ of CellTak (Corning, 354240) for analysis. Concentration of compounds used were 1.5 μM Oligomycin, 6 μM FCCP, 0.5 μM Rotenone/antimycin A, 10 mM glucose, and 50 mM 2-DG.

### Microarray.

TCR transgenic OT-I CD8 T cells (Thy1.1/1.1 or Thy1.1/1.2) cells were injected i.v. into naive Thy1.2 recipients. Mice were immunized 24 hours later with LPS-matured DC coated with OVA peptide (DC-OVA) with or without infection with 2 × 10^5^ PFU LCMV Arm, i.p. At least 1 × 10^6^ OT-I cells were sorted per pool. Alternatively, naive OT-I CD8 T cells were enriched from uninfected donors. RNA was extracted from 3 independent pools of OT-I CD8 T cells from all groups using a RNeasy kit (QIAGEN) according to manufacturer’s instructions. Microarray analysis was performed at the DNA Core facility at the University of Iowa. RNA quality was assessed using the Agilent Model 2100 Bioanalyzer along with the NanoDrop ND-1000. In total, 50 ng RNA was converted to SPIA-amplified cDNA using the WT-Ovation Pico RNA Amplification System, v2 (NuGEN Technologies), according to manufacturer’s instructions. Samples were hybridized onto Affymetrix Mouse Exon 1.0 ST arrays and scanned with the Affymetrix Model 3000 scanner with 7G upgrade. Data were collected using GeneChip operating software (GCOS) v1.4. Data were imported into Partek GS v. 6.12. Normalized log_2_ data were compared using an ANOVA model. Individual comparisons were done using a linear model function. Genes with a *P* value less than 0.01, and a fold-change greater than 1.5 were analyzed further.

### Bulk RNA-seq.

CD8 T cells were isolated and enriched using the EasySep CD8 enrichment kit (StemCell Technologies, 19853). RNA was extracted from CD8 T cells using the RNeasy mini kit (QIAGEN, 74104). RNA concentration and quality were quantified using a NanoDrop 2000c Spectrophotometer. RNA samples were submitted to the IUSM Medical Genomics Sequencing core for analysis. Total RNA samples were first evaluated for their quantity and quality using Agilent Bioanalyzer. In total, 10 ng of RNA was used for library preparation with the SMARTer Stranded Total RNA-Seq Kit v3 -Pico kit (Takara), following the manufacturer’s instruction. Each resulting uniquely dual-indexed library was quantified and quality accessed by Qubit and Agilent TapeStation. Multiple libraries were pooled in equal molarity. The pooled libraries were sequenced on an Illumina NovaSeq X PLUS sequencer. In total, 100 bp paired-end reads were generated.

Reads were mapped to the mouse genome mm10 using STAR (v2.7.10a) (73). RNA-seq aligner with the following parameter: “--outSAMmapqUnique 60”. Uniquely mapped sequencing reads were assigned to Gencode M25 gene using featureCounts (v2.0.1) (74) with the following parameters: “-s 2 –p –Q 10 -O”. The data were filtered using read count > 10 in at least 3 of the samples, normalized using TMM (trimmed mean of M values) method and subjected to differential expression analysis using edgeR (v4.80) (75, 76). Differentially expressed genes (DEGs) were identified using *P* < 0.05 and an absolute log_2_ fold change (|log_2_FC|) > 0.5. DAVID (77, 78) was then used to perform biological enrichment analysis on up- and downregulated DEGs, respectively, including gene ontology (GO) terms and KEGG pathway. The same analysis procedure was applied to published dataset, GSE218660 (55), for gene expression comparison.

### Metabolomics.

CD8 T cells were enriched and activated with anti-CD3 and anti-CD28 as described above. In total, 4 × 10^6^ stimulated CD8 T cells per sample were pelleted, washed with 150 mM ammonium acetate, and flash frozen prior to shipment to the University of Michigan Medical School BRCF Metabolomics Core Facility. Pelleted cell samples were removed from –80°C storage and maintained on ice throughout processing. In total, 500 μL extraction solvent (1:1:1:1, methanol/acetone/acetonitrile/water) was added to the microtube and cells were disrupted using a probe sonicator at 20% output power, 20% duty cycle for 20 seconds. Samples were allowed to rest on ice for 10 minutes and then centrifuged at 4°C, 18,407 x g for 10 minutes. In total, 150 μL of supernatant was transferred to an autosampler vial and brought to complete dryness using a nitrogen drier in ambient conditions. Samples were reconstituted with 25 μL of water: methanol (8:2 by volume).

Ion Pairing Reverse phase LC-MS analysis was performed as previous described (79). Briefly, the system was composed of an Agilent Infinity Lab II UPLC coupled with a 6545 QTof mass spectrometer (Agilent Technologies). The chromatographic separation was performed on an Agilent ZORBAX RRHD Extend 80Å C18, 2.1 × 150 mm, 1.8 μm column with an Agilent ZORBAX SB-C8, 2.1 mm × 30 mm, and 3.5 μm guard column. Mobile phase A consisted of 97:3 water/methanol and mobile phase B was 100% methanol; both A and B contained tributylamine and glacial acetic acid at concentrations of 10 mM and 15 mM, respectively. Glycolysis, TCA, amino acids, and nucleotide metabolites were separated on a 40-minute gradient, and the column was back-flushed with mobile phase C (100% acetonitrile, no additives) between injections for column cleaning.

Semiquantitative data was obtained by manual integration using Profinder v8.00 (Agilent Technologies). Metabolites were identified by matching the retention time (± 0.1 min), mass (± 10 ppm) and isotope profile (peak height and spacing) to authentic standards.

### Statistics.

Statistical analysis was performed using GraphPad Prism software (GraphPad, Version 10.6.1). Coadoptive transfer results were analyzed using a paired 2-tailed Student’s *t* test unless otherwise specified. All other samples were compared with an unpaired Student’s *t* test or 1-way ANOVA with post hoc test for multiple comparisons if there were more than 2 elements being compared. *P* < 0.05 was considered significant.

### Study approval.

Mice were housed, infected, and treated under SPF and BSL-1 or BSL-2 conditions as supervised by the Indiana University IACUC (Protocol no. 23088). Human samples were collected and handled as per IRB approved protocol (protocol no. 16114).

### Data availability.

Data generated are available in the Supporting Data Values file. Microarray data were deposited in the NCBI’s Gene Expression Omnibus (GEO GSE331111). RNA-seq data were deposited in the NCBI’s Gene Expression Omnibus (GEO GSE331107). Metabolomics data are available at the NIH Common Fund’s National Metabolomics Data Repository (NMDR) website, the Metabolomics Workbench (80), https://www.metabolomicsworkbench.org, where it has been assigned Study ID ST004839. The data can be accessed directly via its Project DOI: http://dx.doi.org/10.21228/M81S04

## Author contributions

MEL, SFV, SAC, and MJR conducted experiments, analyzed data, and wrote and edited the manuscript. AEN provided reagents, analyzed data, and edited the manuscript. SL and JW analyzed data and edited the manuscript. MEL and SFV are co–first authors; the authorship order was decided upon mutual agreement based on respective contributions to the manuscript.

## Conflict of interest

The authors have declared that no conflict of interest exists.

## Funding support

This work is the result of NIH funding, in whole or in part, and is subject to the NIH Public Access Policy. Through acceptance of this federal funding, the NIH has been given a right to make the work publicly available in PubMed Central.

- NIH National Institute of Diabetes and Digestive and Kidney Diseases (NIDDK) grant U54 DK106846
- NIH, National Cancer Institute (NCI) grant P30 CA082709
- NIH instrumentation grant 1S10D012270
- NIH grant U2C-DK119886 and OT2-OD030544 grants

## Supplementary Material

Supplemental data

Supporting data values

## Version 1 06/23/2026

In-Press Preview

## Version 2 08/10/2026

Electronic publication
