# Supplementary material for: AFF3 maintains metabolic quiescence in naive CD8 T cells and prevents premature immune aging
Source: JCI Insight. 2026 Jun 23;11(15):e207387. doi: 10.1172/jci.insight.207387 (PMC13464011; doi:10.1172/jci.insight.207387)
Supplement: Supplementary file 1 [file jciinsight-11-207387-s260.pdf]

Lumnitzer et al. Sup. Fig. 1

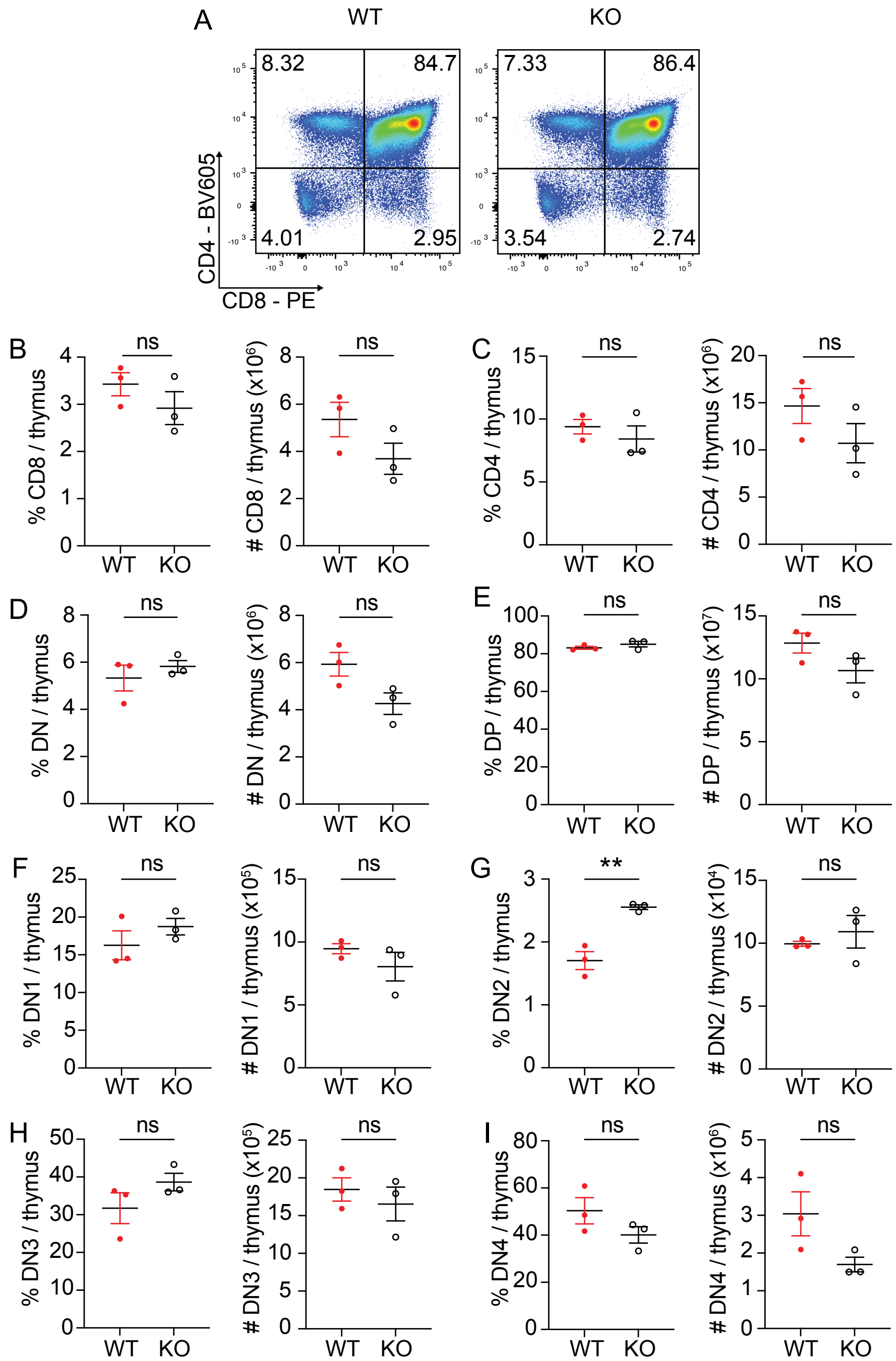


**Supplemental Figure 1.** *Aff3 deletion does not affect T cell development in the thymus.* Thymi from naïve WT and AFF3 KO mice were harvested and analyzed using flow cytometry to compare T cell development. (**A**) Representative flow plot of CD4 and CD8 expression in thymus from naïve WT and AFF3 KO mice showing single positive (SP), double positive (DP), and double negative (DN) (gated on $CD3^-$ cells) T cell populations. (**B, C**) Frequency and number of single positive CD8 T cells and CD4 T cells from the thymi of naive WT or AFF3 KO mice. (**D, E**) Frequency and number of DN and DP T cells from the thymi of naive WT or AFF3 KO mice. (**F-I**) The frequency and number of the four DN populations from the thymi of naive WT or AFF3 KO mice. (A-I) Groups compared using unpaired student T-test, ** <0.01. Representative of 2 experiments with n=3 mice per experiment. Data represent mean ± SEM.

Lumnitzer et al. Sup. Fig. 2

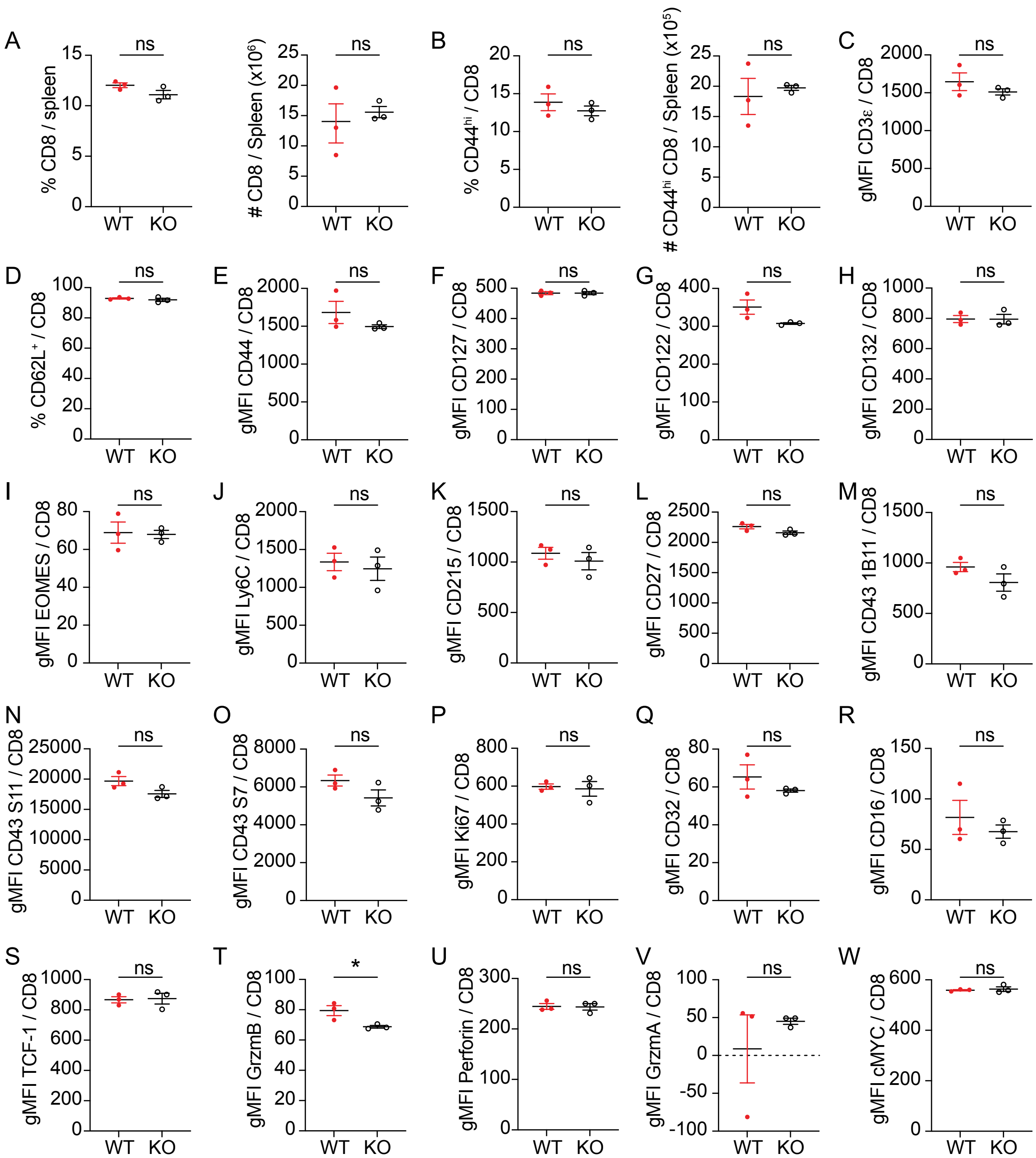


**Supplemental Figure 2.** *AFF3 KO CD8 T cells maintain a naïve phenotype in the spleen.* Spleens from naïve WT and AFF3 KO mice were harvested and analyzed using flow cytometry to compare the phenotype of naïve CD8 T cells. (**A**) Frequency and number of CD8 T cells in the spleen. (**B**) Frequency and number of $CD44^{hi}$ CD8 T cells in the spleen. (**C**) gMFI of $CD3\varepsilon$ expression on the surface of splenic CD8 T cells. (**D**) Frequency of $CD62L^{+}$ CD8 T cells in the spleen. (**E-H, J-O**, **Q-R**) gMFI of the expression of surface proteins on splenic WT and AFF3 KO CD8 T cells, surface protein indicated in the Y axis of the graph. (**I**, **P**, **S-W**) gMFI of the expression of intracellular proteins in WT and AFF3 KO CD8 T cells, protein indicated in the Y axis of the graph. (A-W) Groups compared using unpaired student T-test, * < 0.05. Representative of 2 experiments with n=3 mice per experiment. Data represent mean ± SEM.

Lumnitzer et al. Sup. Fig. 3

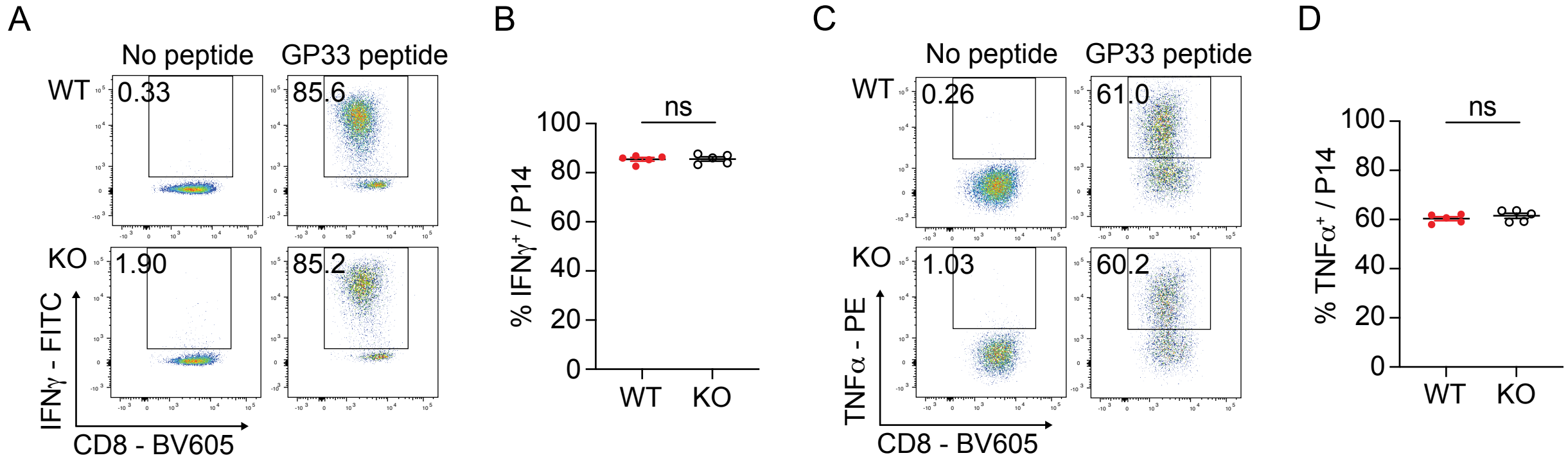


**Supplemental Figure 3.** *The production of cytokines is not different between WT and AFF3 KO CD8 T cells*. (**A, C**) Representative flow plots of intracellular staining for the indicated cytokine by splenic WT or AFF3 KO CD8 T cells with or without stimulation with $GP_{33\text{-}41}$ peptide. Number on the graph represents frequency of cytokine producing cells. (**B, D**) Cumulative frequency of cytokine producing splenic WT or AFF3 KO CD8 T cells. Representative of at least 2 experiments with n=5 mice per group. Groups compared using unpaired student T-test. Data represent mean ± SEM.

Lumnitzer et al. Sup. Fig. 4

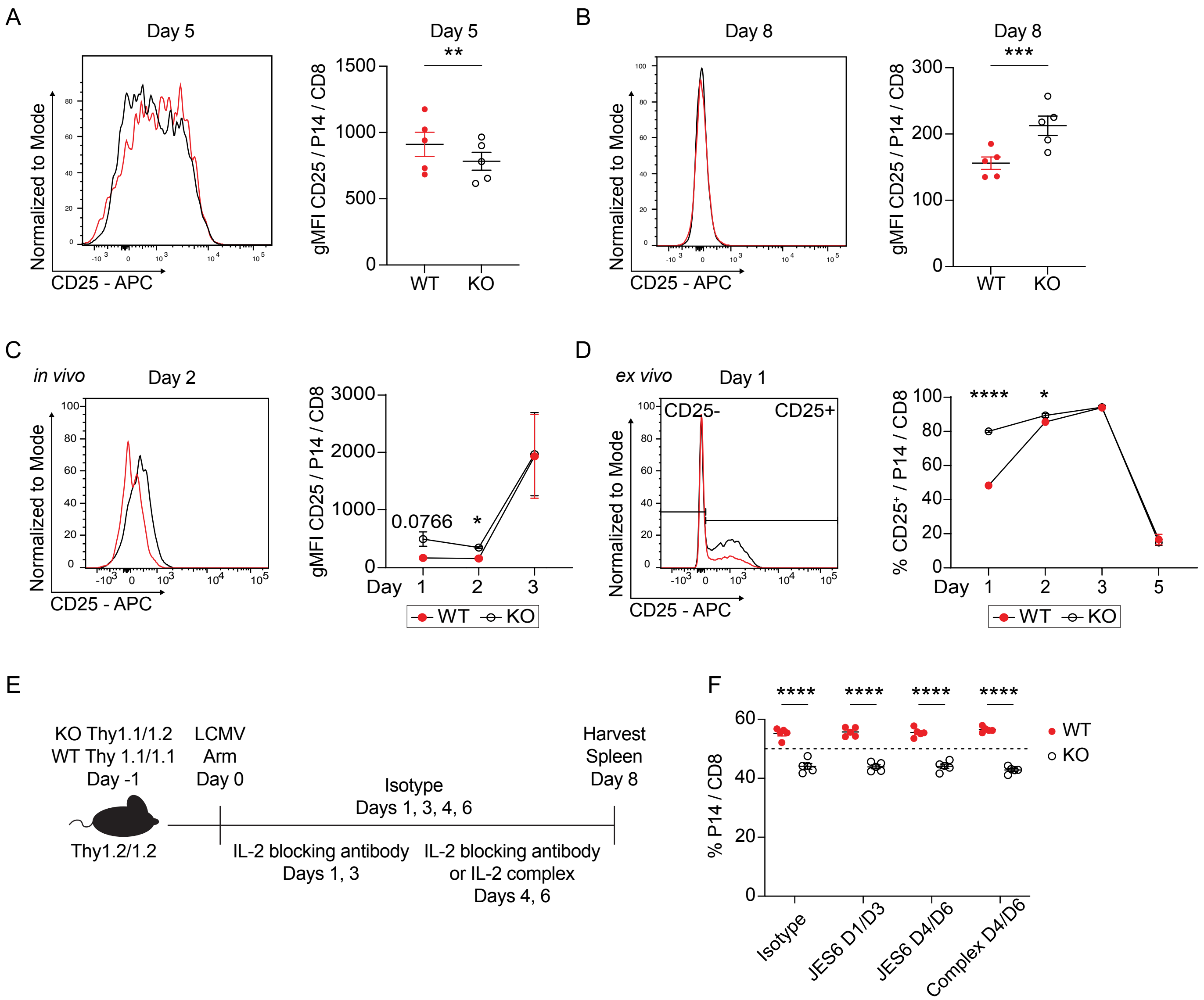


**Supplemental Figure 4.** *The accumulation defect of AFF3 KO CD8 T cells is independent of IL-2.* Naïve C57BL/6 mice received co-transfer of WT and AFF3 KO CD8 T cells, phenotype of the transferred cells was determined after infection. (**A-C**) Representative histograms and quantification by gMFI of CD25 expression on the surface of splenic WT and AFF3 KO CD8 T cells on 5 dpi, 8 dpi, and 1-3 dpi with LCMV Arm. (**D**) Representative histograms and quantification by gMFI of CD25 expression on the surface of splenic WT and AFF3 KO CD8 T cells 1-5 days after ex vivo activation with $GP_{33\text{-}41}$ peptide and anti-CD28 antibody. (**E**) Experimental layout indicating timing of adoptive cell transfer, infection, and treatment of mice. (**F**) Frequency of splenic WT and AFF3 KO P14 CD8 T cells 8 dpi with LCMV Arm, with treatment with anti-IL-2 or IL-2 antibody complexes as indicated. (A-D) Groups compared using unpaired student T-test, p value = 0.05, * < 0.05, ** < 0.01, *** < 0.001, **** < 0.0001. (F) Groups compared using two-way ANOVA analysis with post hoc test, **** < 0.0001. Representative of 2 experiments with n=5 mice per experiment. Data represent mean ± SEM.

Lumnitzer et al. Sup. Fig. 5

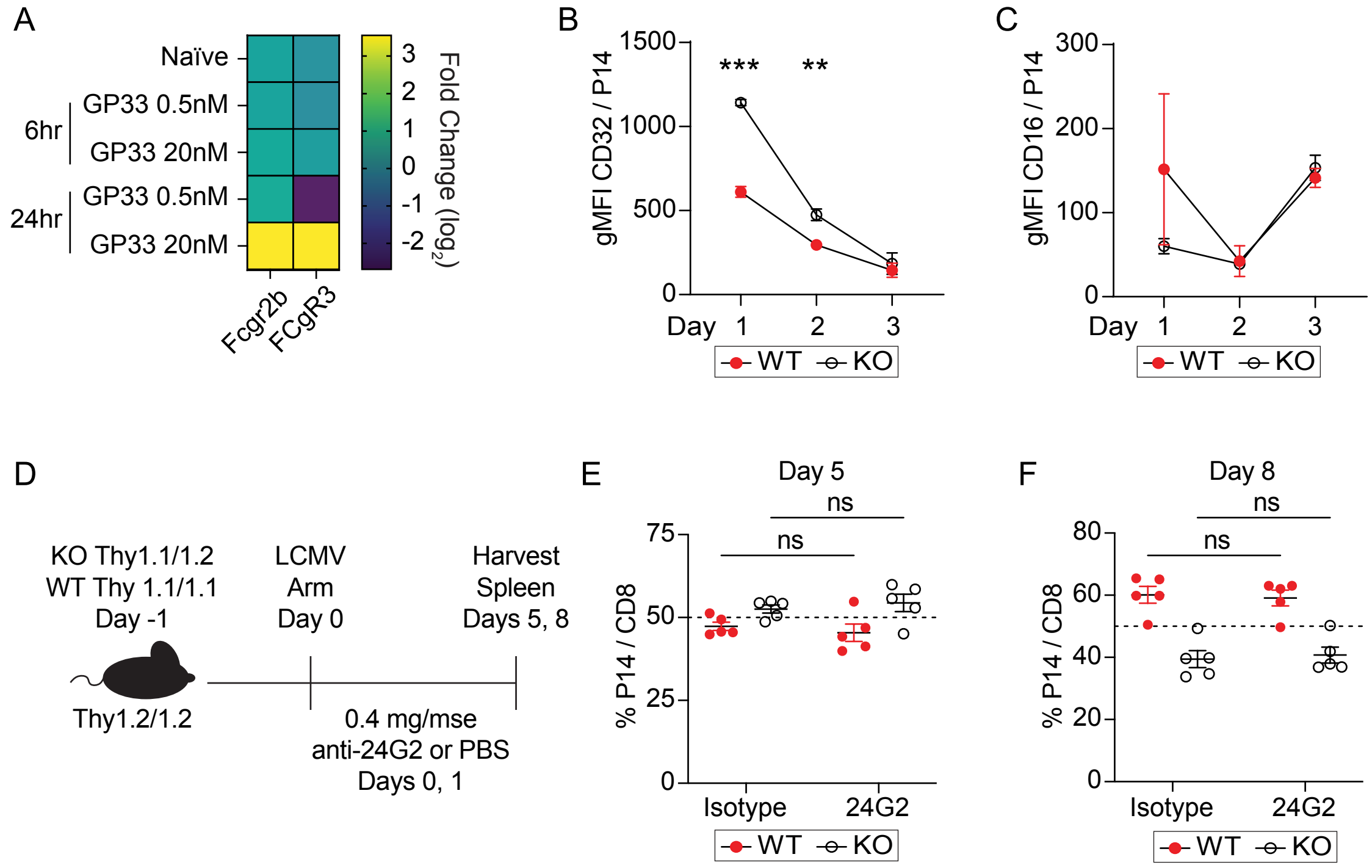


**Supplemental Figure 5.** *The accumulation advantage of the WT CD8 T cell is independent of CD32/CD16.* Based on RNA-seq analysis C57BL/6 mice received co-transfer of WT and AFF3 KO CD8 T cells and treatment with CD16/CD32 blocking antibody, frequency and phenotype of the transferred cells was determined after infection. (**A**) Heat map of *FCGR2B* and *FCGR3A* expression determined by RNA-seq analysis. (**B, C**) Quantification of the gMFI surface expression of CD32 and CD16 (respectively) on splenic WT and AFF3 KO CD8 T cells 1, 2, and 3 dpi with LCMV Arm. (**D**) Schematic showing experimental design with timing of adoptive transfer, infection, and 24G2 antibody treatment. (**E, F**) Frequency of the transferred cells in the isotype control and treated mice at 5 dpi and 8 dpi with LCMV Arm in the spleen. (B-C, E-F). Representative of two experiments with n=5 mice per group. (B,C) Groups compared using paired student T-test. (E-F) Groups compared using two-way ANOVA with post hoc test. ** < 0.01, *** < 0.001. Data represent mean ± SEM.

Lumnitzer et al. Sup. Fig. 6

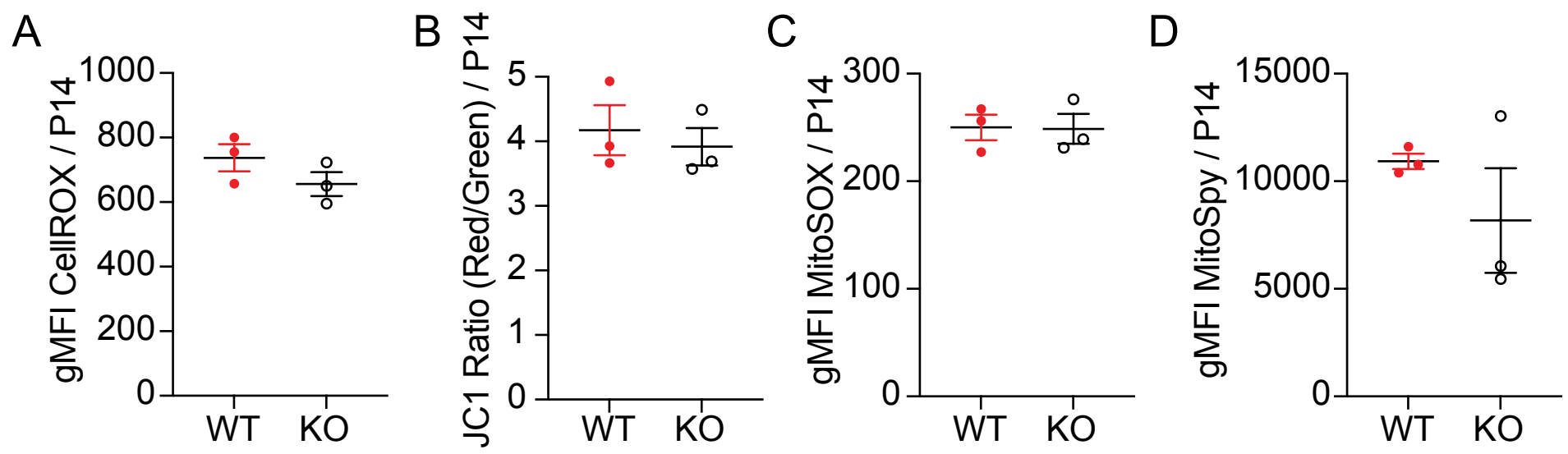


**Supplemental Figure 6.** *AFF3 deficiency does not impact the mitochondrial health of CD8 T cells.* Naïve WT and AFF3 KO CD8 T cells were isolated from the spleen, and their mitochondrial health was determined using flow cytometry. (**A**) Quantification of gMFI of CellROX staining. (**B**) Quantification of ratio of gMFI JC1 polymer/gMFI JC1 monomer indicating membrane polarization. (**C**) Quantification of gMFI of MitoSOX staining. (**D**) Quantification of gMFI of MitoSpy staining. (A-D) Representative of two experiments with n=3 mice per group. Groups compared using unpaired student T-test. Data represent mean ± SEM.

Lumnitzer et al. Sup. Table 1

| Age at time of draw | Sex |
|---|---|
| 29 | Male |
| 28 | Male |
| 25 | Female |
| 22 | Female |
| 25 | Female |
| 27 | Female |
| 32 | Female |
| 33 | Male |
| 25 | Female |
| 63 | Male |
| 66 | Male |
| 65 | Female |
| 60 | Female |
| 68 | Male |
| 66 | Male |
| 61 | Male |
| 65 | Female |

**Supplemental Table 1**. *Information of donors of human PBMC samples.*

## Supplemental methods

*Antibodies.* The antibodies used in this study were as follows in various combination of fluorophores: IFN-$\gamma$ (XMG1.2, BioLegend), TNF-$\alpha$ (MP6-XT22, BioLegend), CD25 (3C7, BioLegend).

*Ex vivo cytokine production.* Splenocytes (1 – 3 x $10^6$) were stimulated with 200nM of $GP_{33-41}$ peptide (Bio-Synthesis) for 5.5 hours at 37$^o$C in the presence of 1x Brefeldin A solution (420601, BioLegend). Cytokines were stained using fluorescently conjugated antibodies via intracellular staining as described in the methods.

*CD32/CD16 blockade.* One day before infection, 10,000 AFF3-KO and WT P14 CD8 T cells were cotransferred at a 1:1 ratio into naive C57BL/6 mice via i.v. injection. Mice were treated on with anti-CD16/CD32 (2.4G2, a gift from Dr. John Harty, University of Iowa) (0.4 mg/mse, i.p.) on 0 and 1 dpi and the frequency of the cells were analyzed on 5 and 8 dpi via flow cytometry.

*IL-2 blockade and supplementation.* One day before infection, 10,000 AFF3-KO and WT P14 CD8 T cells were cotransferred at a 1:1 ratio into naive C57BL/6 mice via i.v. injection. The mice were then given either (0.2 mg/mouse, i.p.) anti-IL-2 (JES6, Bio X Cell) (1 and 3 dpi or 4 and 6 dpi, i.p.) or IL-2 stimulating complex of 50 ug/ml anti-IL-2 (S4B6, Bio X Cell) with 1.5 ug/ml purified mouse IL-2 (Peprotech) (4 and 6 dpi, i.p.). Control mice received injections of isotype control (0.2 mg/mouse, i.p.) (IgG2a$\kappa$ isotype, 2A3, Bio X Cell) on 1, 3, 4, and 6 dpi. Mice were euthanized on 8 dpi and cell frequency was determined using flow cytometry.
